# Following the water? Landscape‐scale temporal changes in bat spatial distribution in relation to Mediterranean summer drought

**DOI:** 10.1002/ece3.4119

**Published:** 2018-05-02

**Authors:** Francisco Amorim, Inês Jorge, Pedro Beja, Hugo Rebelo

**Affiliations:** ^1^ CIBIO‐InBIO, Research Center in Biodiversity and Genetic Resources University of Porto Porto Portugal; ^2^ CEABN‐InBIO Centre for Applied Ecology “Prof. Baeta Neves” Institute of Agronomy University of Lisbon Lisbon Portugal; ^3^ Faculty of Sciences University of Porto Porto Portugal; ^4^ School of Biological Sciences University of Bristol Bristol UK

**Keywords:** acoustic monitoring, habitat use, landscape management, resource tracking, species diversity, water scarcity

## Abstract

Understanding how the spatial distribution of ecological resources shapes species’ diversity and abundance in human‐modified landscapes is a central theme in conservation biology. However, studies often disregard that such patterns may vary over time, thereby potentially missing critical environmental constraints to species persistence. This may be particularly important in highly mobile species such as bats, which are able to track temporal variations in spatial resource distribution. Here we test the hypothesis that bats in Mediterranean landscapes are strongly affected by the progressive reduction in water availability during the seasonal summer drought. We analyzed the effects of landscape composition and structure on bat diversity and activity, during pregnancy, lactation, and postlactation periods, and identified the most influential variables within and across periods. Water bodies showed the strongest positive effect on bats, followed by riparian habitats and areas with steeper (>30%) slopes. However, while during pregnancy, there were only small landscape effects, these increased during lactation and postlactation, highlighting a progressively stronger association with water habitats during the summer drought. The spatial projection of habitat models showed that the landscape distribution of bat diversity and activity hotspots changed markedly over time. During pregnancy, the spatial pattern of hotspot distribution was weakly defined, while during lactation and particularly postlactation, there was a concentration of hotspots along permanently flowing watercourses. Our study highlights that permanently flowing watercourses are critical for bat conservation in Mediterranean landscapes, calling for measures to counteract their ongoing degradation due in particular to climate change, water abstraction and damming. More generally, our study underlines the importance of considering the temporal dimension in habitat selection studies, without which there is the risk of overlooking the importance of habitats that are key for species persistence only at certain times of the year.

## 1 INTRODUCTION

The long‐term persistence of a species in a given landscape is conditional on the availability of resources at the appropriate temporal and spatial scales (Lynch & Ennis, 1983). As the resources and their spatial distribution change over time, it is highly likely that species’ distributions change accordingly to track such resources (Benton, Vickery, & Wilson, 2003). For instance, the food resources required by a species often vary along the life cycle and among life stages (Loureiro, Bissonette, Macdonald, & Santos‐Reis, 2009; Rey, 1995), often associated to spatial changes in food availability. Such changes may be overcome either by species following the resources through different habitats, or by different habitats becoming available at the optimal time for a given species (Benton et al., 2003). Both strategies will lead to temporal variation in species diversity and abundance across the landscape, which should be particularly evident for highly vagile organisms such as bats and birds. Understanding such spatiotemporal patterns is paramount for conservation, due to the need of protecting all habitats providing the resources to fulfill species’ requirements across the whole year (Law & Dickman, 1998). However, such information is seldom available as most studies only provide snapshots from a single season or pool yearly data together into a single analysis, generally disregarding seasonal variations (Bissonette & Storch, 2007; Marra, Cohen, Loss, Rutter, & Tonra, 2015; but see, e.g. Beja et al., 2010; Russell & Ruffino, 2012).

Mediterranean landscapes provide an excellent setting to test hypotheses associated to species resource tracking. This is because the Mediterranean climate is naturally characterized by dry and hot summer periods (Blondel, Aronson, Bodiou, & Boeuf, 2010), and so seasonal water scarcity may strongly determine temporal variations in resource availability. During spring, water availability is usually high, either through precipitation (Magalhães, Beja, Schlosser, & Collares‐Pereira, 2007; Mariotti, Struglia, Zeng, & Lau, 2002) or soil moisture (Miller & Hajek, 1981), which in turn contributes to high levels of photosynthetic activity (Peñuelas, Filella, Llusià, Siscart, & Piñol, 1998) and primary productivity (Melillo et al., 1993). During this season, water also flows in both temporary and permanent water bodies, although this is followed by a declining flow in late spring and subsequent summer drying of watercourses that ends with the first rains of the fall (Gasith & Resh, 1999). Consequently, from late spring to late summer, soil moisture is at its lowest (Miller & Hajek, 1981) and surface water is restricted to the main tributaries, weirs, and dams. This in turn leads to seasonal limitation of plant growth and yield (Flexas et al., 2014; Galmés, Medrano, & Flexas, 2007) and may have consequences for the distributions of invertebrates and vertebrates in general, particularly flying insectivorous vertebrates that may track seasonal variations in resource availability (Bailey et al., 2004; Baxter, Fausch, & Saunders, 2005). Therefore, understanding the responses of insectivorous vertebrates to the seasonal cycle of water availability is critical for conservation in Mediterranean landscapes as it allows identifying the key habitats that need to be maintained to assure sufficient resources throughout the year. This is particularly important given the current and predicted changes to the distribution of water in the Mediterranean due to water abstraction from rivers, large scale construction of dams, and climate changes that are expected to increase the frequency and intensity of summer droughts (Dai, 2011; Hoerling et al., 2012; Milly, Dunne, & Vecchia, 2005; Rebelo & Rainho, 2009).

Bats may be particularly adequate to understand resource tracking in the Mediterranean region because they are flying predators with high mobility, and potentially they respond fast to temporal changes in the spatial distribution of insect prey availability (Power et al., 2004). As a consequence, they may be responsive to the seasonal cycle of water availability, as they are known to be strongly influenced by the availability of aquatic habitats (Salvarina, 2016), particularly in arid and semi‐arid environments (Hagen & Sabo, 2012; Razgour, Korine, & Saltz, 2010). This is supported by studies showing that ponds in Mediterranean forests have higher bat activity and diversity than the adjacent areas of the forest matrix (Lisón & Calvo, 2014), and that permanent water bodies and riparian habitats are important for both bat species diversity and activity (Rainho, 2007; Razgour, Hanmer, & Jones, 2011; Russo & Jones, 2003). There is also evidence that small artificial water bodies such as farm dams may be beneficial to bats (Sirami, Jacobs, & Cumming, 2013; Tuttle, Chambers, & Theimer, 2006), often attracting species that are widespread and abundant across the landscape (Hintze et al., 2016; Lisón & Calvo, 2011). Furthermore, water availability seems to have strong effects on the condition and reproductive output of individuals (Adams & Hayes, 2008; Amorim, Mata, Beja, & Rebelo, 2015), further stressing the importance to adjust habitat use to the availability of water resources. Despite these observations, there is still limited understanding on how bat distributions vary over time in Mediterranean landscapes, and it remains uncertain how these changes may be driven by temporal variations in the distribution of water resources (but see, e.g., Salvarina, Gravier, & Rothhaupt, 2018; Dalhoumi, Morellet, Aissa, & Aulagnier, 2017).

Here we tested the hypothesis that bats in Mediterranean landscapes are strongly affected by the progressive reduction in water availability during the seasonal summer drought. For that purpose, we evaluated changes in habitat use and the spatial distribution of both species richness and activity during bats’ active phase (from spring to autumn). Our specific aims were as follows: (1) to determine which habitat variables are associated to species richness and bat activity considering three key phenological periods (pregnancy, lactation, and postlactation); (2) to estimate whether the importance of habitat variables varied across the phenological periods; and (3) to estimate temporal variations in the spatial distribution of bat species richness and activity hotspots. We predict that the spatial distribution of bat diversity and activity should be largely independent of water availability in spring, during pregnancy, but as summer progresses bats should be progressively more constrained by the spatial distribution of the remnant surface waters (Adams, 2010; Adams & Hayes, 2008).

## 2 MATERIALS AND METHODS

### 2.1 Study area

**Figure 1:**
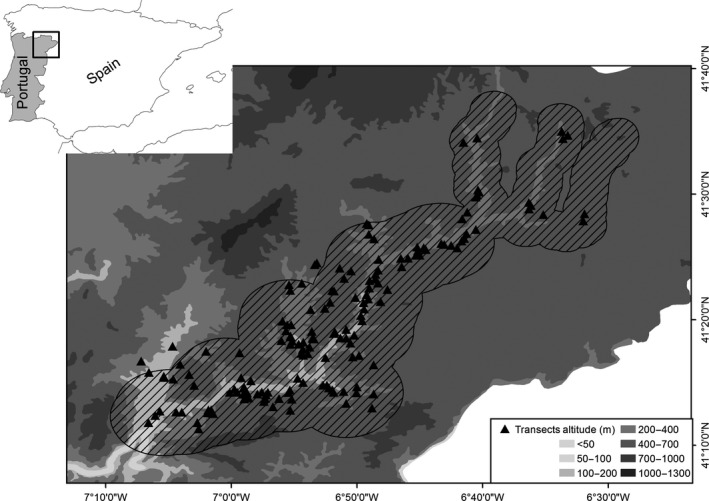
Study area (line filled) in northeastern Portugal and location of the acoustic transects (*n* = 155) sampled for bats July to October 2011, and from May to September 2012

The study was carried out in northeast Portugal (41°21′0″N, 6°58′0″W), within the Baixo Sabor Long Term Ecological Research Site (LTER_EU_PT_002). Specifically, we mainly focused on a 1,100 km^2^ area defined by a 5‐km buffer around the main river and a 2‐km buffer around its main tributaries (Figure 1), because we were interested in documenting bat activity relatively close to the main waterlines, and that could thus be more influenced by the seasonal changes in surface water availability. The region is in the transition between meso‐ and supra‐Mediterranean bioclimatic zones, with cold winters (average temperature of the coldest month <6°C) and dry summers (total annual precipitation <600 mm, of which <5% in July–August), which are particularly hot in some valleys where monthly average temperatures exceed 21°C (Monteiro‐Henriques, 2010). The landscape is characterized by plateaus at about 700–800 m a.s.l., which are dissected by deep and narrow streams valleys. Land cover is dominated by a complex mosaic of natural vegetation patches, forest stands (mainly maritime pine *Pinus pinaster* plantations), permanent crops (mainly olive and almond groves), and pastures, which reflect a process of progressive agricultural land abandonment since the 1960s (Hoelzer, 2003). Natural vegetation is largely composed of shrublands of variable structure and species composition, remnants of native evergreen oak woodlands, and some well‐developed riparian galleries (*Quercus suber*,* Q. rotundifolia*) (Hoelzer, 2003). Primary productivity peaks in winter and early spring, while the lowest values are observed in summer (Amorim et al., 2015).

### 2.2 Study design

The study was based on acoustic surveys carried out along 200 transects from July to October 2011, and from May to September 2012. Transects were distributed in the study area using a stratified randomization, in order to have a comparable sampling effort across the dominant land cover types. Each transect was surveyed only once during the study, in either the pregnancy (May–June), lactation (July–August), or postlactation (September–October) periods. These time windows cover the corresponding phenological period for most European bat species (Amorim et al., 2015; Goiti, Aihartza, Almenar, Salsamendi, & Garin, 2006; Pretzlaff, Kerth, & Dausmann, 2010; Racey & Swift, 1985). The option to sample different transects in different periods was taken to maximize the coverage of environmental variability in the study area, under the logistic constraints limiting the maximum number of transects that could be sampled per period. However, temporal comparability of results was assured by sampling the same geographic areas, and by maintaining a similar representation of each land cover type across sampling periods. We used data from each phenological period to build seasonal habitat models, and pooled data across periods to build an annual habitat model. These models were then used to predict the distribution of species richness and bat activity across the landscape, for each time period.

### 2.3 Bat acoustic surveys

Bats were sampled using acoustic surveys, which started 45 min after sunset and lasted for three hours, corresponding to the period of highest bat activity (Duffy, Lumsden, Caddle, Chick, & Newell, 2000; Rainho, 2007; Russo & Jones, 2003; Vaughan, Jones, & Harris, 1997; Wickramasinghe, Harris, Jones, & Vaughan, 2003). Sampling was always made by the same observer (FM), accompanied by a second person. We only sampled during nights with favorable weather conditions for bat activity, specifically with no rain, low humidity, mild temperature, and null or weak wind (Amorim, Rebelo, & Rodrigues, 2012; Russo & Jones, 2003). However, sometimes weather changed during a given night or at specific locations, and so the corresponding transects were discarded. Each transect was walked at low speed (ca. 2 km/h) for 15 min, and all bat activity was recorded using a handheld ultrasound detector (D1000X; Pettersson Elektronik AB, Uppsala, Sweden) with a sampling frequency of 384 kHz. Species were identified using sound‐analysis software (BatSound Pro 4.2, Pettersson Elektronik AB, Uppsala, Sweden) with a 1024 pt FFT and Hamming window for spectrogram analysis (Amorim, Carvalho, Honrado, & Rebelo, 2014; Russo & Jones, 2003). Acoustic identification of bat calls was based on Russo & Jones, 2002; Pfalzer & Kusch, 2003; Walters et al., 2012; Rainho, Alves, & Marques, 2013. Bat calls that could not be assigned to a species or species group were considered as nonidentified calls and were only considered to estimate overall bat activity. Bat activity was measured at 10‐s intervals.

### 2.4 Landscape predictors

We estimated variables describing landscape composition (land cover type) and structure (topography and configuration metrics), within a 500‐m buffer around each sampling transect (Table S1). This radius was chosen considering previous studies showing that bat presence at a site is highly influenced by habitat features within 100‐500 m (Bellamy, Scott, & Altringham, 2013). All variables were extracted from digital thematic layers using QGIS 2.18.4 (QGIS Development Team, 2017) and the following R packages: rgdal (Bivand, Keitt, & Rowlingson, 2016), maptools (Bivand & Lewin‐Koh, 2016), raster (Hijmans, 2016), and sp (Bivand, Pebesma, & Gomez‐Rubio, 2013; Pebesma & Bivand, 2005). Topographic variables were estimated using a 25‐m resolution digital elevation model (http://www.eea.europa.eu/dataand-maps/data/eu-dem). For each buffer, we computed the maximums, minimums, means, medians, ranges and standard deviations of elevation, slope and aspect. In addition, we estimated the proportion of the buffer occupied by high slopes, using 20º, 30º, and 40º as alternative thresholds. Slopes were considered because they are expected to affect bats, as they provide roosting opportunities (Santos et al., 2014), are used as landmark during commuting and foraging (Russo, Cistrone, & Jones, 2005), and may even assist bats to perform ascending flights while foraging (Roeleke, Bumrungsri, & Voigt, 2018). Land cover variables were extracted from the Portugal's digital Land Cover Map of 2007 (http://www.igeo.pt/) and were quantified as the proportion within the buffers of land cover classes aggregated into nine main categories judged a priori to reflect contrasting bat habitats (Rainho, 2007; Rebelo & Rainho, 2009): Mediterranean forest, riparian habitat, shrublands, water bodies, orchards, arable lands, conifers, eucalyptus plantations, urban areas (Table S1). We only considered permanent water bodies, most of which are natural in the study area. Landscape structure was quantified by first reclassifying the land cover classes into “open” and “closed” habitats, and then computing patch richness, median patch area, and edge density metrics computed with Fragstats 4.2 (McGarigal, Cushman, & Ene, 2012). Urban areas and closed and mixed forests were classified as “closed” habitats, while open forests, shrublands, water bodies, arable land, and orchards were classified as “open” habitats (Table S1). This reclassification was judged to provide a better description of landscape heterogeneity for bats than the original land cover classes, because echolocation limits the range of habitat structures a bat can explore and forage, leading to open or cluttered vegetation adaptation (Neuweiler, 1989).

### 2.5 Data analysis

Prior to habitat modeling, we assessed collinearity among variables by computing Variance Inflation Factors (VIF), and we dropped from subsequent analysis all variables with VIF > 7. In addition, we computed pairwise Pearson's correlations (*r*) between variables, and dropped one variable from each pair showing *r* > 0.7. Finally, we inspected the histograms of variables for excess of zeros and outliers, and dropped the urban and eucalypt land cover classes due to their very low representation. These procedures reduced the environmental variables used in analysis from 50 to 22 (Table 1). Regarding the response variables, we confirmed that they were not spatially autocorrelated using spline correlogram plots with 95% pointwise confidence intervals calculated with 1,000 bootstrap resamples (BjØrnstad & Falck, 2001) (Figure S1), thereby indicating that autocorrelation did not contribute to biases in estimates of model coefficients and significance levels (Diniz‐Filho, Rangel, & Bini, 2008; Rhodes, McAlpine, Zuur, Smith, & Ieno, 2009).

**Table 1:** Description and summary statistics (mean values and standard deviation) of landscape composition and structure variables used to model bat species richness and total activity in northeastern Portugal

Landscape composition	Description	Mean ± *SD*
Mediterranean forest	Proportion of Mediterranean forest in 500‐m buffer	0.17 ± 0.16
Riparian habitat	Proportion riparian habitat in 500‐m buffer	0.01 ± 0.01
Shrublands	Proportion of shrublands in 500‐m buffer	0.36 ± 0.22
Water bodies	Proportion of water bodies in 500‐m buffer	0.02 ± 0.03
Orchards	Proportion of orchards in 500‐m buffer	0.28 ± 0.21
Arable lands	Proportion of arable land in 500‐m buffer	0.06 ± 0.13
Landscape structure		
Altitude		
Standard deviation	Altitude standard deviation	49.78 ± 20.38
Slope		
Median	Median slope	54.08 ± 2.89
Slope area		
>30º	Proportion of buffer area with slope higher than 30º	0.05 ± 0.08
Northness (aspect cosine)		
Median	Median northness	0.02 ± 0.03
Eastness (aspect sine)		
Median	Median eastness	0.03 ± 0.03
Number of closed patches	Number of land cover patches classified as closed weighted by total buffer area	0.03 ± 0.03
Area of open patches	Mean area of land cover patches classified as open weighted by total buffer area	0.06 ± 0.04
Edge density of closed patches	Edge density of land cover patches classified as closed weighted by total buffer area	0.01 ± 0.03
Closed patches richness	Number of land cover categories classified as closed weighted by total buffer area	0.01 ± 0.01
Number of open patches	Number of land cover patches classified as open weighted by total buffer area	0.16 ± 0.07
Edge density of open patches	Edge density of land cover patches classified as open weighted by total buffer area	0.79 ± 0.32
Area of closed patches	Mean area of land cover patches classified as closed weighted by total buffer area	0.04 ± 0.06
Open patches richness	Number of land cover categories classified as open weighted by total buffer area	0.03 ± 0.01

Seasonal and annual habitat relations were estimated using generalized linear models, with Poisson's distribution and log link function for species richness, and negative binomial distribution and log link function for bat activity. No correction for overdispersion was needed for species richness models, while the negative binomial models adequately accounted for high overdispersion in bat activity data (Ver Hoef & Boveng, 2007). Models were built separately for the landscape composition and structure sets of variables, because combining the two might obscure the effects of landscape structure given the strong affinities of bats for particular habitat types (Russo & Jones, 2003). Model building was based on the model selection and averaging procedure of Burnham and Anderson (2002), which compares the relative support of a suite of candidate models using Akaike's information criterion (AIC) and the corresponding Akaike weights (*w*_*i*_). Candidate models were built using all possible combinations of variables, and model building involved a two‐step procedure. For each landscape model, we computed an average model based on the 95% confidence set of candidate models, and estimated the sum of the Akaike weights (*w*_*i*_+) as a measure of its relative importance in the model. Variables with a probability of selection above 0.65 were then carried out to the second model building step, where we repeated the model selection and averaging procedure. Inferences were made considering the selection probability of each explanatory variable along with the uncertainty in parameter estimates with 95% confidence intervals (95% CI), with variables with CI overlapping zero considered to have equivocal meaning (Burnham & Anderson, 2002).

Analyses were performed in R 3.3.2 (R Core Team, 2016), using the usdm package (Naimi, 2015) to compute VIFs, the MASS package (Venables & Ripley, 2002) for generalized linear modeling, and the MuMIn package (Barton, 2016) for model selection and averaging.

### 2.6 Species richness and bat activity mapping

The seasonal habitat models were projected into the study area to identify hotspots of bat species richness and activity. To do this, we first created a hexagonal grid covering the whole study area (Birch, Oom, & Beecham, 2007), with hexagon area similar to that of the median transect buffer (c.a.109.21 m^2^). The environmental variables were extracted for each polygon using the procedure described above and then we used the habitat models to predict the species richness (number of species per 15‐min interval) and bat activity (bat passes/min) for each polygon. Hotspots of species richness corresponded to hexagons with >3.5 species per 15‐min interval, whereas hotspots of bat activity correspond to hexagons with >2 bat passes/min. Seasonal maps were then overlapped, and the consistency in hotspot location across seasons was estimated and depicted with Venn diagrams built using Venneuler R package (Wilkinson, 2011).

## 3 RESULTS

### 3.1 Acoustic surveys

From the initial 200 transects sampled during a total of 50 h, only 155 provided data with sufficient quality for subsequent analysis, due to low recording quality resulting for instance from equipment malfunction or background noise, local adverse weather conditions, and other field constraints such as terrain ruggedness. From these, 51 were sampled during pregnancy, 60 during lactation, and 44 during postlactation. A total of 6929 bat passes were recorded, of which 66% (4551) could be identified to species level, yielding a total of 19 species or species groups. Pipistrelle species had the highest activity levels, with *Pipistrellus pipistrellus* representing 47% of the identified bat passes, followed by *P. kuhlii* (17%), *Tadarida teniotis* (17%), and *Myotis daubentonii* (13%). A total of 327 (7%) bat passes were assigned to the small *Myotis* group (*M. daubentonii*,* M. emarginatus*,* M. mystacinus*,* M. bechsteinii,* and *M. escalerai*), although in most cases these probably belong to *M. daubentonii* that could not be reliably identified due to poor recording quality.

### 3.2 Species richness

**Figure 2:**
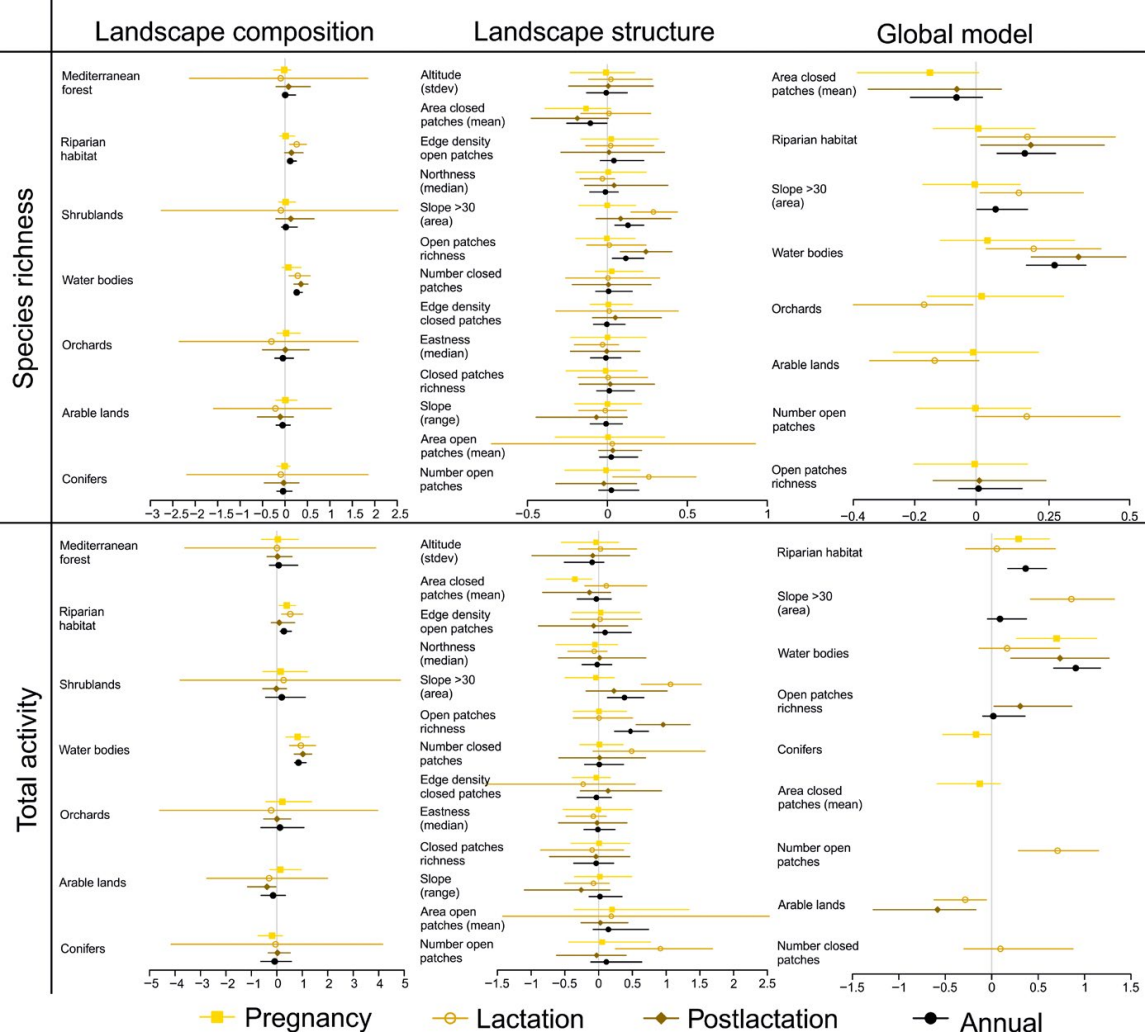
Forest plots summarizing average models relating bat species richness and total activity to either landscape composition, landscape structure, or a combination of landscape composition and structure (global) variables in northeast Portugal. Different models were built for each phenological period (pregnancy—filled square, lactation—empty square, postlactation—filled diamond) and for data combined over the annual cycle (Annual—filled circle). For each average model, we plot the regression coefficient estimates and the corresponding 95% confidence interval for each variable included in the model. Details of each model are provided in Tables S2 and S3

Landscape composition models provided moderate (0.9 > *w*_*i*_ + >0.7) to high (*w*_*i*_ + >0.9) support for positive effects of cover by riparian habitats and water bodies on species richness in all phenological periods except pregnancy (Figure 2, Table S2). The negative effects of orchards were moderately supported, but only during lactation. Regarding landscape structure, there was moderate‐to‐high support for the positive effect of steeper slope (>30%) areas, more patches of open habitats, and higher richness of open patches, and for the negative effect of the mean size of closed habitat patches, but the effects were inconsistent across periods (Figure 2, Table S2). Global models combining composition and structure variables suggested that species richness was mainly affected by landscape composition, with consistently positive effects of riparian cover and water bodies, except during pregnancy when there was no effect was supported (Figure 2, Table S2). During lactation, there was moderately supported positive effects of steeper slope (>30º) areas and the number of open patches, and negative effects of orchards and arable land cover. The effect of steeper slopes was also moderately supported in the annual model.

### 3.3 Bat activity

Landscape composition models provided high support for the positive effects of cover by riparian habitats (except in postlactation) and water bodies in all phenological periods and in the annual model, while the negative effect of conifer plantations was only moderately supported during pregnancy (Figure 2, Table S3). Landscape structure models provided moderate‐to‐high support for higher bat activity in areas of steeper slopes (>30%), with more patches of open habitats, and higher richness of open patches, and negative effects of the mean patch size of closed habitats (Figure 2, Table S3), but effects were inconsistent across periods. Global models provided moderate‐to‐high supported for positive effects of water bodies in all periods except lactation and over the annual cycle, riparian habitats during pregnancy and over the annual cycle, and for steeper slope areas and number of open patches during lactation (Figure 2, Table S3). There was also a moderately supported negative effect of arable land during lactation.

The modeling procedure for total bat activity was repeated after excluding data for *P. pipistrellus*, because this species represented 65% of the identified bat passes and could thus have a strong influence in the habitat associations uncovered. Likewise, we removed nonidentified calls, because 80% of these were assigned to species groups including *P. pipistrellus*. The new landscape composition models provided strong support for the positive effects of water bodies, and moderate support for the negative effects of arable land, in all phenological periods except pregnancy and over the annual cycle (Table S4). The landscape structure models provided high support for the effects of steeper slope (>30º) areas and the number of closed patches during lactation, for open patch richness during postlactation, and for steeper slope areas and open patch richness over the annual cycle (Table S4). Other variables showed only moderate support, and their effects were inconsistent across periods.

### 3.4 Hotspots of bat species richness and activity

**Figure 3:**
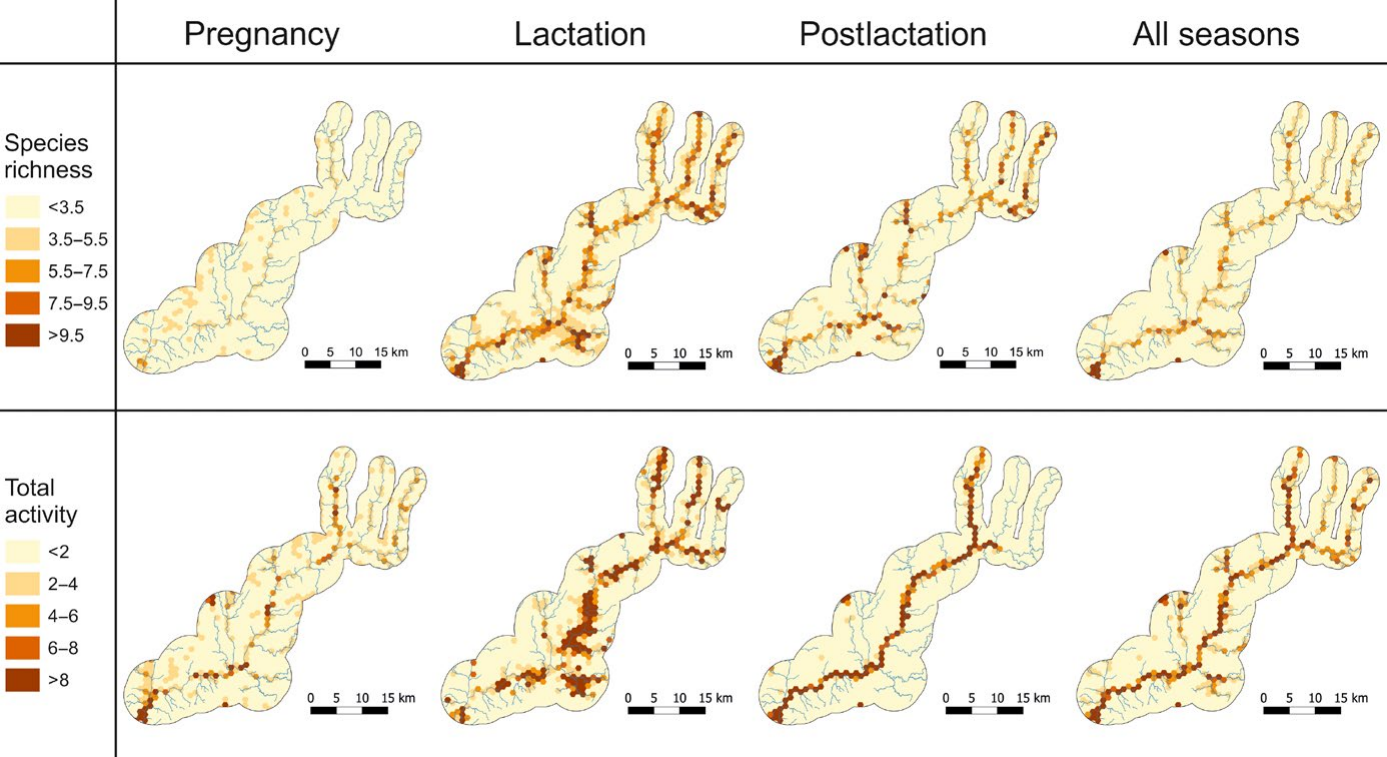
Spatial distribution of hotspots (hexagons) of bat species richness (>3.5 species per 15‐min interval) and total activity (>2 bat passes/min) in northeastern Portugal, estimated from the spatial projection of the global landscape models provided in Tables S2 and S3. Separate maps are provided for each phenological period (pregnancy, lactation, and postlactation) and for data combined over the annual cycle

**Figure 4:**
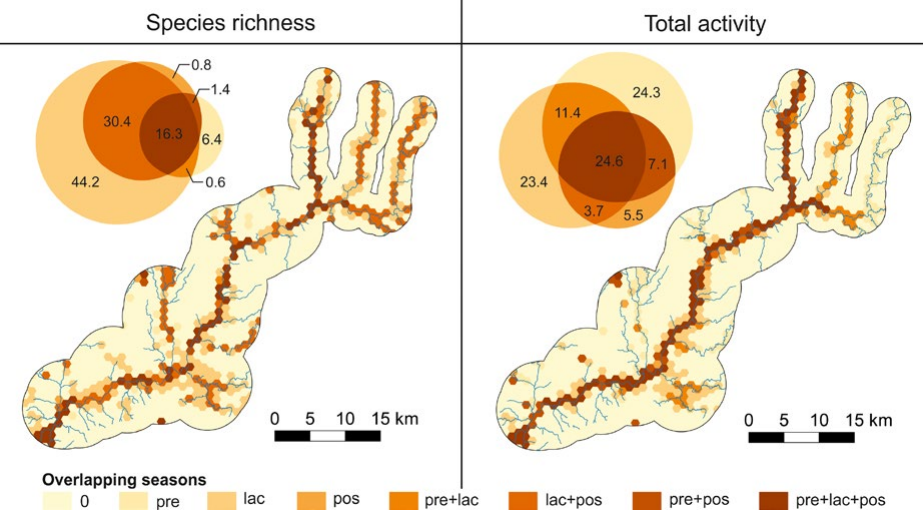
Spatial overlap in the distribution of hotspots (hexagons) of bat species richness (>3.5 species per 15‐min interval) and total activity (>2 bat passes/min) in northeastern Portugal, across the three phenological periods considered in the study (Pre—pregnancy, Lac—lactation, Pos—postlactation). Zero represents areas without bat hotspots in any phenological period, while the remaining colors represent overlaps between different combinations of phenological periods. Venn diagrams shows the percentage overlap of hotspots among the three phenological periods

During pregnancy, there was no obvious pattern in the spatial distribution of species richness hotspots, while during lactation and postlactation, there was a clear concentration of hotspots along the main river and its two largest tributaries (Figure 3). The hotspots of bat activity were similar to those of species richness and were always concentrated along the main river and its two largest tributaries, although this pattern was much weaker during pregnancy than during lactation and postlactation (Figure 3). There were also important differences between the two latter periods, with activity hotspots during lactation occurring all along the main river valley and its tributaries, while during postlactation it was concentrated almost exclusively in a narrow strip along the main river (Figure 3). Predictions considering the entire breeding season also identified the same areas as hotspots of bat activity, although the spatial patterns were less‐well defined than during either the lactation or postlactation periods (Figure 3). Overall, there was a large temporal mismatch between the spatial distribution of hotspots, with only 16.3% and 24.6% being common across the three breeding periods for richness and activity, respectively (Figure 4). Most of these consistent hotspots are located along the main river (Figure 4). Spatial projection of the standard error can be found in Figure S2.

## 4 DISCUSSION

As predicted, our results have identified a seasonal pattern in habitat preferences of bats in a Mediterranean landscape of northeastern Portugal, suggesting that bats may track the spatiotemporal dynamics of water availability. Overall, species richness and bat activity were mainly shaped by the habitats where water was available (water bodies, riparian galleries), with the strength of such associations peaking at the end of summer, when surface waters were mainly available in large streams and rivers (Ferreira, Filipe, Bardos, Magalhães, & Beja, 2016). In contrast, in spring, during pregnancy, bats did not show strong associations to specific habitat features, probably due to higher water availability across the landscapes. Overall, our results point out the need to understand how vagile species such as bats modify their habitat associations and spatial distribution over the annual cycle, which is essential to determine the habitats that are needed year round to assure species persistence (Bissonette & Storch, 2007).

### 4.1 Limitations and potential shortcomings

Our study had some limitations and potential shortcomings, but we believe that they did not affect our key results in any significant way. First, although our sample size was relatively small, the number of transects surveyed was comparable to that of similar studies (e.g., Davy, Russo, & Fenton, 2007; Mendes, Fonseca, Marques, Maia, & Ramos Pereira, 2017; Rainho, 2007; Salvarina et al., 2018; Vaughan et al., 1997), and it was sufficient to detect 19 of the 25 bat species occurring in continental Portugal. Therefore, it is unlikely that small sample sizes were responsible for the strong associations observed with water bodies and riparian habitats, or the marked variations in the spatial distribution of species richness and bat activity. Second, it should be considered that each transect was sampled only once, and so the sampling network varied across years and phenological periods. The sampling design was chosen to incorporate as much environmental variability as possible, while overcoming logistic limitations that prevented us from sampling every site during each period. This strategy is not without potential problems, however, as it might be argued that the patterns observed could be due to the sampling of different areas at different times of the year. We believe this is unlikely to have biased the results, because in each season we randomly distributed the transects across the study area, and stratified sampling so that at least three transects representative of each land cover type were visited in each season. In this way, we avoided time × space and time × habitat interactions that could have affected our results. Finally, our results on bat activity may be dominated by the spatial patterns of a single species, *P. pipistrellus*, which was by far the most frequently recorded. It should be noted, however, that our results for bat activity were largely consistent with those of obtained with species richness, although the later variable should not be influenced by the abundance of a single species. Also, models developed after excluding *P. pipistrellus* still provided high support for the importance of water bodies, particularly during the lactation and postlactation periods, although the effects of environmental variables in general were much less supported.

### 4.2 Water is a key landscape feature for bats

We found a strong positive association between bats and habitats where water is available (water bodies and riparian galleries), which was evident in analysis based on either species richness or total activity, and that was largely supported in most phenological periods and over the annual cycle. The association with these habitats had consequences for the landscape‐scale distribution of bats, with species richness and activity often peaking close to large rivers and streams. Reasons for these patterns are uncertain, but they may reflect the abundance of prey close to water bodies (Fukui, Murakami, Nakano, & Aoi, 2006; Goiti, Garin, Almenar, Salsamendi, & Aihartza, 2008; Hagen & Sabo, 2012; Lisón, López‐Espinosa, Calvo, & Jones, 2015; Salvarina et al., 2018), the need to drink water (Adams & Hayes, 2008; Greif & Siemers, 2010; Russo & Jones, 2003; Tuttle et al., 2006), or a combination of these and other ecological factors. Whatever the causes, the importance of aquatic habitats for bats has been reported in a large number of studies (review in Salvarina, 2016), including studies carried out in the Mediterranean region. For instance, Russo and Jones (2003) showed that water sites corresponded to the habitat most used by bats, while a large number of endangered or vulnerable species occurred in riparian habitats, broad‐leaved woodlands, and olive groves. Also, Rainho (2007) found that water sites during the summer period supported high species richness, while riparian habitats surrounded by autochthonous broad‐leaved forests provided optimal foraging areas. Finally, Lisón and Calvo (2013) showed using ecological niche modeling that pipistrelle species have a strong preference for aquatic habitats, while a telemetry study by Salsamendi et al. (2012) concluded that *Rhinolophus mehelyi* foraged close to water bodies, where it was judged to have access to drinking water and higher insect abundances. Comparable patterns were found in other regions, with particularly strong associations between bats and water reported in arid and semi‐arid environments, including for instance the Middle East, North Africa, and parts of North America (Hagen & Sabo, 2014; Korine, Adams, Russo, Fisher‐Phelps, & Jacobs, 2016; Razgour et al., 2010; Rebelo & Brito, 2007).

The other landscape variables considered in our study had much weaker effects, and these were often inconsistent across phenological periods. One of the variables showing the most supported positive effects was the area with steeper slopes (>30%), but this may also reflect the presence of deep river valleys and thus the proximity to water and riparian galleries. However, this variable may also reflect the presence of bat roosts in cliffs and other steep areas (Santos et al., 2014). This is supported by the strongest effect of steep slopes on both species richness and activity during lactation, a period when lactating females have smaller home ranges, fly shorter distances, and return to roosts more often during the night, leading to increased activity near roosts (Henry, Thomas, Vaudry, & Carrier, 2002; Lučan & Radil, 2010). Still weaker and more inconsistent effects were found for variables that describe landscape structure such as open patches richness, number of open patches and mean area of closed patches and the presence of edges, which are related to landscape heterogeneity, and may thus affect bat diversity and activity (Jantzen & Fenton, 2013; Stein, Gerstner, & Kreft, 2014). However, the effect of these variables may only be perceived at fine spatial scales, which may explain their modest contribution in our study.

### 4.3 Water resource tracking by bats in Mediterranean landscapes

Our results indicate that the effect of water bodies on bat species richness and activity increased consistently over the breeding season, and there was a progressive spatial concentration of diversity and activity hotspots close to permanently flowing waters. In fact, while in springtime, during pregnancy, there were neither strong habitat effects or marked spatial patterns of hotspot distribution, in late summer, during postlactation, there were very strong effects of water bodies and the hotspots were distributed along the largest river in the region. These results suggest that bats may track spatial variations in water availability, which in the Mediterranean is at its highest in spring and at its lowest in late summer (Gasith & Resh, 1999; Magalhães et al., 2007). This is also the case in our study area, where water availability progressively decreases during the summer, with smaller tributaries and upper reaches drying out, and surface waters remaining primarily in the main river and the largest tributaries (Ferreira et al., 2016).

The reason for bats tracking the receding waters is unknown, but it may be a consequence of the changes in the availability of critical resources during the summer, coupled with changes in the requirements of individuals during the breeding season. One possibility is that insect prey is highest close to water bodies during the dry season, in a period of low primary productivity throughout most of the landscape (Amorim et al., 2015). In fact, water availability is known to affect insect prey distribution and abundance (Bailey et al., 2004; Hawkins & Porter, 2003), and in summer the emergence of adult insects from streams may offer feeding opportunities for bats (Baxter et al., 2005; Fukui et al., 2006; Hagen & Sabo, 2012). Also, the availability of drinking water may be low throughout most of the landscape, which may constrain bats to areas close to the main rivers and streams where they can access this important resource (Adams & Hayes, 2008; Greif & Siemers, 2010; Russo & Jones, 2003; Tuttle et al., 2006). These factors may explain why there was no marked effects during the spring of water bodies, or actually of any habitat feature, as water availability and primary productivity tend to be high across the landscape. Furthermore, pregnant females may range more widely and forage for longer periods than during lactation and postlactation (Henry et al., 2002; Encarnação, Dietz, & Kiedrorf, 2004; Daniel, Korine, & Pinshow, 2010; but see, Vincent, Nemoz, & Aulagnier, 2011), thereby having the ability to move over large areas and thus presumably having weaker associations with specific habitat features. In contrast, during lactation females have the highest energetic demands (Anthony & Kunz, 1977; Dietz & Kalko, 2006; Kurta, Kunz, & Nagy, 1990), which may constrain their foraging activity to areas with high prey availability close to roosts, thereby justifying the increased association with water bodies and riparian galleries. Clearly, these issues require further investigation to understand the extent to which the spatiotemporal tracking of water availability is a general pattern in dry landscapes, and to identify the factors driving such resource tracking. For this, it is important to develop more studies characterizing habitat associations over the breeding season, because the usual practice of pooling all data into a single yearly dataset (Russo & Jones, 2003; Salsamendi et al., 2012) cannot detect eventual seasonal patterns in bat habitat use.

### 4.4 Conservation implications

Our results show that the habitat associations and distribution of bats in Mediterranean landscapes may change over the annual cycle, with species richness and activity progressively concentrating throughout the summer in the few habitats where water remains available. This has important consequences for conservation, as bat breeding season in the Mediterranean partly overlaps with the peak of dry conditions (Altringham, 1996; Amorim et al., 2015; Audet, 1990; Ibáñez, 1997; Racey & Swift, 1985; Rodrigues, Zahn, Rainho, & Palmeirim, 2003), thus reductions in water availability may reduce reproductive success compromising species persistence (Adams, 2010; Adams & Hayes, 2008; Amorim et al., 2015; Safi & Kerth, 2004). In fact, reduction in the availability of surface waters during the summer may decrease the opportunities for drinking (Korine et al., 2016; Rainho, 2007), reduce prey availability (Hagen & Sabo, 2012; Salvarina et al., 2018), and increase competition among individuals at remnant waters (Adams, Pedersen, Thibault, Jadin, & Petru, 2003; Razgour, Korine, & Saltz, 2011). Bats may thus be strongly affected by current trends of climate change, as the predicted increases in the frequency and severity of summer droughts in the Mediterranean region will likely reduce water flows (Milly et al., 2005), thereby degrading habitat suitability for bats during critical periods. Increasing damming of rivers for hydroelectric power generation and to feed irrigated agriculture is also likely to reduce flowing waters and thus habitat suitability for bats (Rebelo & Rainho, 2009), although small‐scale artificial bodies of water may promote bat diversity and activity in arid environments (Razgour et al., 2010; Sirami et al., 2013; Tuttle et al., 2006). Finally, the degradation of water quality due to pollution may further reduce the availability of suitable water habitats for bats (Korine, Adams, Shamir, & Gross, 2015; Salvarina, 2016; Vaughan, Jones, & Harris, 1996).

Overall, our results point out that rivers and larger streams that maintain water through the dry season should be considered a priority for bat conservation in the Mediterranean region, thereby further supporting the need to maintain their ecological integrity for a range of both aquatic and terrestrial species (Baxter et al., 2005; Carvalho, Brito, Crespo, & Possingham, 2010; Gasith & Resh, 1999; Matos, Santos, Palomares, & Santos‐Reis, 2009; Rebelo & Rainho, 2009). More generally, our results support the value of considering the temporal dimension of habitat studies, as ignoring spatiotemporal heterogeneities in resource use and availability may contribute for overlooking critical habitats for species persistence in dynamic landscapes (Bissonette & Storch, 2007).

## CONFLICT OF INTEREST

None declared.

## AUTHORS’ CONTRIBUTIONS

FA, PB, and HR initiated the study. FA performed the field work and collected the data. FA and IJ organized the database, did GIS‐work and statistical analyses. FA drafted the manuscript with main input from PB and HR. All authors edited and approved the final manuscript.

## DATA ACCESSIBILITY

Data available from the Dryad Digital Repository: https://doi.org/10.5061/dryad.44n7b7t.
