# Supplementary figures and images for: Following the water? Landscape‐scale temporal changes in bat spatial distribution in relation to Mediterranean summer drought
Source: Ecol Evol. 2018 May 2;8(11):5801–14. doi: 10.1002/ece3.4119 (PMC6010748; doi:10.1002/ece3.4119)

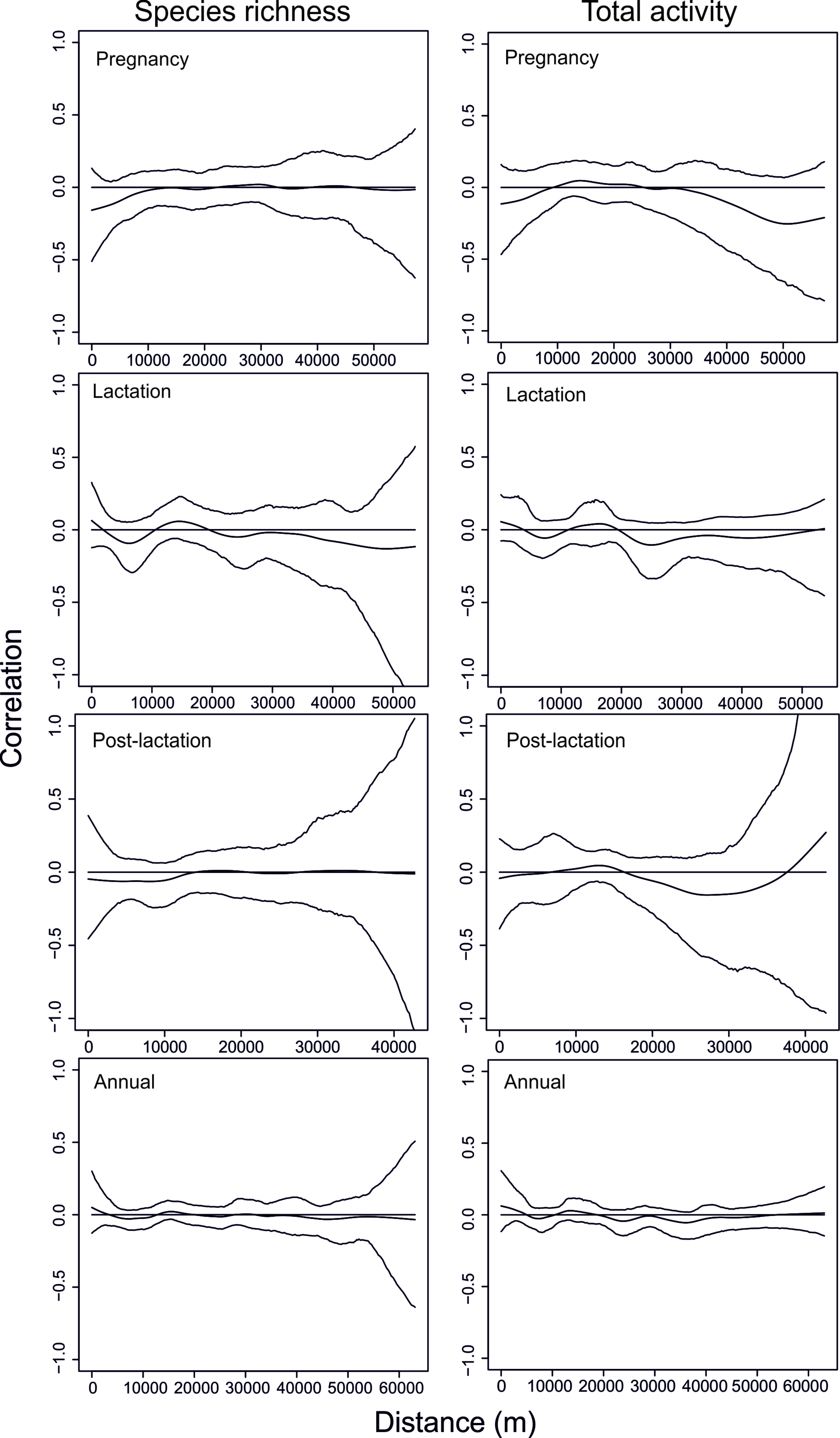

Supplement: Supplementary file 1 [file ECE3-8-5801-s001.tiff]

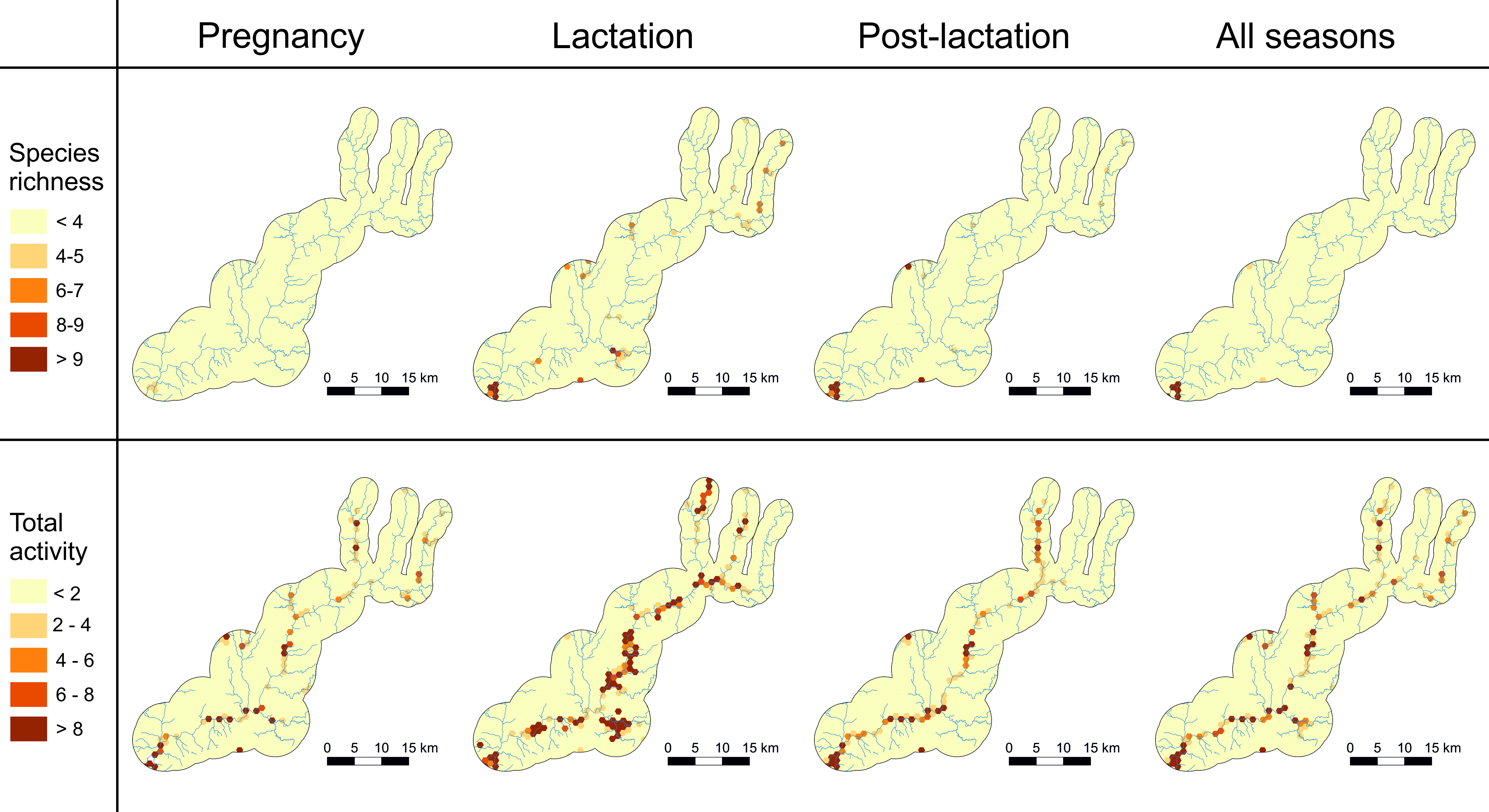

Supplement: Supplementary file 2 [file ECE3-8-5801-s002.tiff]
