# Supplementary material for: Following the water? Landscape‐scale temporal changes in bat spatial distribution in relation to Mediterranean summer drought
Source: Ecol Evol. 2018 May 2;8(11):5801–14. doi: 10.1002/ece3.4119 (PMC6010748; doi:10.1002/ece3.4119)
Supplement: Supplementary file 3 [file ECE3-8-5801-s003.docx]

**Table S1**- Landscape composition and structure variables used to model bat habitats in NE Portugal. For each variable we provide a short description, summary statistics (Mean ± Sandard Deviation [SD]) and, where appropriate, its reclassification as “open” (O) or “closed” (C) according to habitat clutter to calculate configuration metrics.

| **Variables** | **Description** | **Mean ± SD** | **Clutter** |
| --- | --- | --- | --- |
| **Landscape composition** |  |  |  |
| Mediterranean forest | Proportion of Mediterranean forest in 500-m buffer | 0.17 ± 0.16 | C/O^a^ |
| Riparian habitat | Proportion riparian habitat in 500-m buffer | 0.01 ± 0.01 | C |
| Shrublands | Proportion of shrublands in 500-m buffer | 0.36 ± 0.22 | O |
| Water bodies | Proportion of water bodies in 500-m buffer | 0.02 ± 0.03 | O |
| Orchards | Proportion of orchards in 500-m buffer | 0.28 ± 0.21 | O |
| Arable land | Proportion of arable land in 500-m buffer | 0.06 ± 0.13 | O |
| Conifers | Proportion of conifer plantations in 500-m buffer | 0.09 ± 0.15 | C/O^a^ |
| Eucalyptus plantations | Proportion of Eucalyptus plantations in 500-m buffer | 0.01 ± 0.04 | C/O^a^ |
| Urban areas | Proportion of urban areas in in 500-m buffer | 0.00 ± 0.00 | C |
| **Landscape structure** |  |  |  |
| Altitude  Standard deviation | Altitude standard deviation | 49.78 ± 20.38 |  |
| Slope  Median | Median slope | 54.08 ± 2.89 |  |
| Slope area  >20º  >30º  >40º | Proportion of buffer area with slope higher than 20º  Proportion of buffer area with slope higher than 30º  Proportion of bufferarea with slope higher than 40º | 0.28 ± 0.22  0.05 ± 0.08  0.00 ± 0.01 |  |
| Northness (aspect cosine)  Median | Median northness | 0.02 ± 0.03 |  |
| Eastness (aspect sine)  Median | Median eastness | 0.03 ± 0.03 |  |
| Number of closed patches | Number of land cover patches classified as closed weighed by total buffer area | 0.03 ± 0.03 |  |
| Area of open patches | Mean area of land cover patches classified as open weighed by total buffer area | 0.06 ± 0.04 |  |
| Edge density of closed patches | Edge density of land cover patches classified as closed weighed by total buffer area | 0.01 ± 0.03 |  |
| Closed patches richness | Number of land cover categories classified as closed weighed by total buffer area | 0.01 ± 0.01 |  |
| Number of open patches | Number of land cover patches classified as open weighed by total buffer area | 0.16 ± 0.07 |  |
| Edge density of open patches | Edge density of land cover patches classified as open weighed by total buffer area | 0.79 ± 0.32 |  |
| Area of closed patches | Mean area of land cover patches classified as closed weighed by total buffer area | 0.04 ± 0.06 |  |
| Open patches richness | Number of land cover categories classified as open weighted by total buffer area | 0.03 ± 0.01 |  |

^a^ Forest patches (Mediterranean forest, Conifers and Eucalyptus plantations) were classified as either “closed” (closed and mixed forest) or “open” (open forest and agroforestry systems)

**Figure S1 –** Spline correlograms describing spatial autocorrelation in bat species richness and total activity. Separate correlograms are presented for each phenological period (Pregnancy, Lactation, Post-lactation) and over the annual cycle. Lines represent the estimate (in the middle) and the 95% confidence envelopes (external lines) using 1000 bootstrap resamples.

**Table S2 –** Summary results of average models relating bat species richness to landscape composition and structure variables. Models were built separately for each phenological period (Pregnancy, Lactation, Post-Lactation) and for data aggregated across periods (Annual). In each case, separate models were also built for landscape composition, landscape structure, and the combination of landscape composition and structure variables. For each variable we provide the estimate of the regression coefficient (B), its standard error (SE) and 95% confidence interval (95%CI), the sum of Akaike weights (*w_i_*+), the number of models in which the variable was included (*N*), and the significance level. *** *P* < 0.001; ** *P* < 0.01; * *P* < 0.05. Variables with *w_i_*+ >0.70 are highlighted in bold

**Landscape composition models**

**Pregnancy**

| Variables | B | SE | 95% CI | *w_i_*+ | *N* | *P* |
| --- | --- | --- | --- | --- | --- | --- |
| (Intercept) | 1.152 | 0.087 | [9.77E-01, 1.33E+00] |  |  | <2.0E-16 *** |
| Mediterranean forest | -0.018 | 0.058 | [-2.52E-01, 1.30E-01] | 0.29 | 38 | 0.765 |
| Riparian habitat | 0.015 | 0.051 | [-1.19E-01, 2.18E-01] | 0.30 | 42 | 0.777 |
| Shrublands | 0.014 | 0.054 | [-1.35E-01, 2.33E-01] | 0.29 | 39 | 0.797 |
| Water bodies | 0.074 | 0.104 | [-6.40E-02, 3.56E-01] | 0.50 | 50 | 0.485 |
| Orchards | 0.025 | 0.080 | [-1.77E-01, 3.43E-01] | 0.30 | 39 | 0.762 |
| Arable land | 0.007 | 0.061 | [-2.06E-01, 2.63E-01] | 0.26 | 37 | 0.905 |
| Conifers | -0.009 | 0.040 | [-1.76E-01, 1.12E-01] | 0.27 | 37 | 0.830 |

**Lactation**

| Variables | B | SE | 95% CI | *w_i_*+ | *N* | *P* |
| --- | --- | --- | --- | --- | --- | --- |
| (Intercept) | 1.261 | 0.089 | [1.08E+00, 1.44E+00] |  |  | < 2.0E-16 *** |
| Mediterranean forest | -0.067 | 0.694 | [-2.13E+00, 1.85E+00] | 0.47 | 16 | 0.924 |
| **Riparian habitat** | **0.290** | **0.093** | **[1.04E-01, 4.75E-01]*** | **1** | **27** | **2.2E-03 **** |
| Shrublands | -0.061 | 0.945 | [-2.76E+00, 2.51E+00] | 0.5 | 16 | 0.949 |
| **Water bodies** | **0.314** | **0.131** | **[8.88E-02, 5.62E-01]*** | **0.96** | **26** | **1.8E-02*** |
| **Orchards** | **-0.273** | **0.884** | **[-2.36E+00, 1.63E+00]** | **0.75** | **17** | **0.761** |
| Arable land | -0.184 | 0.551 | [-1.59E+00, 1.03E+00] | 0.66 | 13 | 0.741 |
| Conifers | -0.064 | 0.629 | [-2.19E+00, 1.85E+00] | 0.38 | 14 | 0.920 |

**Post-lactation**

| Variables | B | SE | 95% CI | *w_i_*+ | *N* | *P* |
| --- | --- | --- | --- | --- | --- | --- |
| (Intercept) | 0.947 | 0.113 | [7.19E-01, 1.18E+00] |  |  | < 2.0E-16 *** |
| Mediterranean forest | 0.080 | 0.155 | [-1.97E-01, 5.62E-01] | 0.44 | 26 | 0.611 |
| **Riparian habitat** | **0.143** | **0.124** | **[-7.01E-03, 4.03E-01]** | **0.72** | **32** | **0.254** |
| Shrublands | 0.129 | 0.196 | [-2.05E-01, 6.50E-01] | 0.58 | 28 | 0.517 |
| **Water bodies** | **0.355** | **0.078** | **[1.98E-01, 5.12E-01]*** | **1** | **53** | **9.1E-06 ***** |
| Orchards | 0.006 | 0.153 | [-5.00E-01, 5.35E-01] | 0.34 | 23 | 0.970 |
| Arable land | -0.105 | 0.175 | [-6.14E-01, 1.85E-01] | 0.49 | 29 | 0.555 |
| Conifers | -0.030 | 0.126 | [-4.63E-01, 3.10E-01] | 0.39 | 26 | 0.816 |

**Annual**

| Variables | B | SE | 95% CI | *w_i_*+ | *N* | *P* |
| --- | --- | --- | --- | --- | --- | --- |
| (Intercept) | 1.109 | 0.047 | [1.02E+00, 1.20E+00] |  |  | < 2.0E-16 *** |
| Mediterranean forest | 0.045 | 0.069 | [-5.85E-02, 2.34E-01] | 0.52 | 14 | 0.513 |
| **Riparian habitat** | **0.156** | **0.048** | **[6.04E-02, 2.51E-01]*** | **1** | **29** | **1.4E-03 **** |
| Shrublands | 0.057 | 0.083 | [-7.72E-02, 2.75E-01] | 0.57 | 15 | 0.499 |
| **Water bodies** | **0.299** | **0.044** | **[2.13E-01, 3.86E-01]*** | **1** | **29** | **< 2.0E-16 ***** |
| Orchards | -0.008 | 0.066 | [-2.29E-01, 1.88E-01] | 0.38 | 14 | 0.906 |
| Arable land | -0.014 | 0.049 | [-1.96E-01, 1.14E-01] | 0.33 | 13 | 0.782 |
| Conifers | -0.004 | 0.047 | [-1.78E-01, 1.55E-01] | 0.30 | 13 | 0.940 |

**Landscape structure models**

**Pregnancy**

| Variables | B | SE | 95% CI | *w_i_*+ | *N* | *P* |
| --- | --- | --- | --- | --- | --- | --- |
| (Intercept) | 1.145 | 0.083 | [9.77E-01, 1.31E+00] |  |  | <2.0E-16 *** |
| Altitude (stdev) | -0.008 | 0.054 | [-2.32E-01, 1.71E-01] | 0.27 | 1763 | 0.880 |
| **Area closed patches (mean)** | **-0.133** | **0.120** | **[-3.90E-01, 2.15E-02]** | **0.72** | **2510** | **0.275** |
| Edge density open patches | 0.025 | 0.077 | [-1.65E-01, 3.18E-01] | 0.32 | 1897 | 0.754 |
| Northness (median) | 0.006 | 0.056 | [-1.96E-01, 2.43E-01] | 0.26 | 1690 | 0.917 |
| Slope >30 (area) | 1.2E-5 | 0.045 | [-1.78E-01, 1.78E-01] | 0.26 | 1685 | 1 |
| Open patches richness | -0.003 | 0.047 | [-1.96E-01, 1.73E-01] | 0.26 | 1679 | 0.95 |
| Number closed patches | 0.027 | 0.058 | [-7.64E-02, 2.24E-01] | 0.37 | 1904 | 0.64 |
| Edge density closed patches | 0.007 | 0.036 | [-1.06E-01, 1.57E-01] | 0.28 | 1763 | 0.85 |
| Eastness (median) | 0.002 | 0.060 | [-2.29E-01, 2.43E-01] | 0.26 | 1672 | 0.977 |
| Closed patches richness | -0.010 | 0.061 | [-2.58E-01, 1.86E-01] | 0.28 | 1735 | 0.872 |
| Slope (range) | 0.001 | 0.054 | [-2.04E-01, 2.14E-01] | 0.27 | 1733 | 0.98 |
| Area open patches (mean) | 0.004 | 0.087 | [-3.26E-01, 3.58E-01] | 0.26 | 1684 | 0.962 |
| Number open patches | -0.008 | 0.062 | [-2.65E-01, 2.03E-01] | 0.27 | 1701 | 0.897 |

**Lactation**

| Variables | B | SE | 95% CI | *w_i_*+ | *N* | *P* |
| --- | --- | --- | --- | --- | --- | --- |
| (Intercept) | 1.091 | 0.083 | [9.24E-01, 1.26E+00] |  |  | < 2.0e-16 *** |
| Altitude (stdev) | 0.027 | 0.069 | [-1.17E-01, 2.81E-01] | 0.33 | 930 | 0.698 |
| Area closed patches (mean) | 0.015 | 0.063 | [-1.65E-01, 2.72E-01] | 0.28 | 904 | 0.813 |
| Edge density open patches | 0.026 | 0.071 | [-1.35E-01, 2.89E-01] | 0.33 | 1087 | 0.721 |
| Northness (median) | -0.026 | 0.047 | [-1.72E-01, 4.67E-02] | 0.41 | 1090 | 0.5854 |
| **Slope >30 (area)** | **0.293** | **0.073** | **[1.47E-01, 4.38E-01]** | **1** | **2215** | **8.2e-05 ***** |
| Open patches richness | 0.017 | 0.057 | [-1.29E-01, 2.42E-01] | 0.30 | 953 | 0.770 |
| Number closed patches | 0.009 | 0.077 | [-2.61E-01, 3.27E-01] | 0.26 | 884 | 0.910 |
| Edge density closed patches | 0.016 | 0.102 | [-3.21E-01, 4.44E-01] | 0.27 | 876 | 0.876 |
| Eastness (median) | -0.025 | 0.053 | [-2.06E-01, 7.10E-02] | 0.37 | 974 | 0.643 |
| Closed patches richness | 0.009 | 0.059 | [-1.84E-01, 2.52E-01] | 0.28 | 921 | 0.876 |
| Slope (range) | -0.008 | 0.041 | [-1.79E-01, 1.19E-01] | 0.28 | 901 | 0.844 |
| Area open patches (mean) | 0.036 | 0.252 | [-7.24E-01, 9.27E-01] | 0.35 | 1148 | 0.888 |
| **Number open patches** | **0.265** | **0.152** | **[3.43E-02, 5.55E-01]** | **0.90** | **1642** | **0.086 .** |

**Post-lactation**

| Variables | B | SE | 95% CI | *w_i_*+ | *N* | *P* |
| --- | --- | --- | --- | --- | --- | --- |
| (Intercept) | 1.100 | 0.097 | [9.04E-01, 1.30E+00] |  |  | <2E-16 *** |
| Altitude (stdev) | 0.006 | 0.070 | [-2.42E-01, 2.87E-01] | 0.28 | 1256 | 0.930 |
| **Area closed patches (mean)** | **-0.188** | **0.142** | **[-4.76E-01, 6.42E-03]** | **0.80** | **1865** | **0.195** |
| Edge density open patches | 0.011 | 0.092 | [-2.90E-01, 3.58E-01] | 0.31 | 1409 | 0.910 |
| Northness (median) | 0.042 | 0.095 | [-1.42E-01, 3.78E-01] | 0.36 | 1329 | 0.666 |
| Slope >30 (area) | 0.083 | 0.116 | [-6.98E-02, 3.98E-01] | 0.51 | 1544 | 0.481 |
| **Open patches richness** | **0.241** | **0.081** | **[8.11E-02, 4.04E-01]** | **1** | **2964** | **3.82e-3 **** |
| Number closed patches | 0.008 | 0.065 | [-2.19E-01, 2.73E-01] | 0.28 | 1265 | 0.911 |
| Edge density closed patches | 0.050 | 0.091 | [-9.22E-02, 3.37E-01] | 0.41 | 1355 | 0.587 |
| Eastness (median) | -0.004 | 0.056 | [-2.31E-01, 2.03E-01] | 0.27 | 1259 | 0.949 |
| Closed patches richness | 0.018 | 0.070 | [-1.76E-01, 2.95E-01] | 0.31 | 1272 | 0.799 |
| Slope (range) | -0.068 | 0.121 | [-4.46E-01, 1.24E-01] | 0.42 | 1387 | 0.581 |
| Area open patches (mean) | 0.034 | 0.059 | [-5.51E-02, 2.15E-01] | 0.43 | 1464 | 0.569 |
| Number open patches | -0.022 | 0.078 | [-3.22E-01, 1.82E-01] | 0.32 | 1291 | 0.780 |

**Annual**

| Variables | B | SE | 95% CI | *w_i_*+ | *N* | *P* |
| --- | --- | --- | --- | --- | --- | --- |
| (Intercept) | 1.126 | 0.047 | [1.03E+00, 1.22E+00] |  |  | <2.0E-16 *** |
| Altitude (stdev) | -0.001 | 0.034 | [-1.29E-01, 1.25E-01] | 0.28 | 985 | 0.984 |
| **Area closed patches (mean)** | **-0.100** | **0.077** | **[-2.53E-01, -2.64E-03]** | **0.78** | **1444** | **0.197** |
| Edge density open patches | 0.047 | 0.067 | [-4.37E-02, 2.28E-01] | 0.51 | 1314 | 0.487 |
| Northness (median) | -0.006 | 0.026 | [-1.10E-01, 6.75E-02] | 0.28 | 907 | 0.817 |
| **Slope >30 (area)** | **0.135** | **0.049** | **[4.79E-02, 2.28E-01]** | **0.98** | **2081** | **0.006 **** |
| **Open patches richness** | **0.122** | **0.057** | **[3.19E-02, 2.28E-01]** | **0.94** | **1801** | **0.035 *** |
| Number closed patches | 0.014 | 0.039 | [-7.25E-02, 1.56E-01] | 0.34 | 1029 | 0.719 |
| Edge density closed patches | 0.003 | 0.026 | [-8.60E-02, 1.11E-01] | 0.27 | 897 | 0.899 |
| Eastness (median) | -0.003 | 0.025 | [-1.05E-01, 8.42E-02] | 0.26 | 877 | 0.915 |
| Closed patches richness | 0.019 | 0.044 | [-6.57E-02, 1.69E-01] | 0.36 | 1031 | 0.669 |
| Slope (range) | -0.002 | 0.026 | [-1.06E-01, 9.41E-02] | 0.26 | 871 | 0.951 |
| Area open patches (mean) | 0.031 | 0.053 | [-4.69E-02, 1.89E-01] | 0.43 | 1047 | 0.563 |
| Number open patches | 0.032 | 0.055 | [-5.32E-02, 1.97E-01] | 0.44 | 1058 | 0.568 |

**Global models**

**Pregnancy**

| Variables | B | SE | 95% CI | *w_i_*+ | *N* | *P* |
| --- | --- | --- | --- | --- | --- | --- |
| (Intercept) | 1.155 | 0.086 | [9.81E-01, 1.33E+00] |  |  | <2e-16 *** |
| Area closed patches (mean) | -0.150 | 0.117 | [-3.87E-01, 8.26E-03] | 0.79 | 101 | 0.204 |
| Riparian habitat | 0.007 | 0.045 | [-1.39E-01, 1.93E-01] | 0.27 | 69 | 0.874 |
| Slope >30 (area) | -0.004 | 0.041 | [-1.73E-01, 1.44E-01] | 0.26 | 64 | 0.927 |
| Water bodies | 0.037 | 0.082 | [-1.16E-01, 3.21E-01] | 0.36 | 75 | 0.656 |
| Orchards | 0.018 | 0.066 | [-1.59E-01, 2.85E-01] | 0.29 | 70 | 0.786 |
| Arable land | -0.009 | 0.063 | [-2.70E-01, 2.03E-01] | 0.27 | 62 | 0.89 |
| Number open patches | -0.002 | 0.047 | [-1.95E-01, 1.79E-01] | 0.25 | 62 | 0.967 |
| Open patches richness | -0.004 | 0.047 | [-2.02E-01, 1.68E-01] | 0.25 | 62 | 0.927 |

**Lactation**

| Variables | B | SE | 95% CI | *w_i_*+ | *N* | *P* |
| --- | --- | --- | --- | --- | --- | --- |
| (Intercept) | 1.184 | 0.088 | [1.01E+00, 1.36E+00] |  |  | < 2.0E-16 *** |
| **Riparian habitat** | **0.171** | **0.139** | **[5.36E-03, 4.53E-01]** | **0.75** | **13** | **0.223** |
| **Slope >30 (area)** | **0.143** | **0.105** | **[1.34E-02, 3.49E-01]** | **0.79** | **16** | **0.176** |
| **Water bodies** | **0.192** | **0.115** | **[3.34E-02, 4.07E-01]** | **0.87** | **16** | **0.097.** |
| **Orchards** | **-0.165** | **0.119** | **[-4.00E-01, -1.07E-02]** | **0.81** | **12** | **0.170** |
| **Arable land** | **-0.131** | **0.105** | **[-3.46E-01, 9.08E-03]** | **0.78** | **13** | **0.219** |
| **Number open patches** | **0.169** | **0.145** | **[-2.26E-03, 4.69E-01]** | **0.72** | **14** | **0.247** |

**Post-lactation**

| Variables | B | SE | 95% CI | *w_i_*+ | *N* | *P* |
| --- | --- | --- | --- | --- | --- | --- |
| (Intercept) | 0.995 | 0.101 | [7.91E-01, 1.20E+00] |  |  | < 2E-16 *** |
| Area closed patches (mean) | -0.063 | 0.099 | [-3.51E-01, 8.27E-02] | 0.47 | 3 | 0.536 |
| **Riparian habitat** | **0.179** | **0.122** | **[1.56E-02, 4.17E-01]** | **0.82** | **4** | **0.151** |
| **Water bodies** | **0.334** | **0.077** | **[1.80E-01, 4.88E-01]** | **1** | **6** | **2.2E-05 ***** |
| Open patches richness | 0.011 | 0.049 | [-1.40E-01, 2.28E-01] | 0.24 | 2 | 0.831 |

**Annual**

| Variables | B | SE | 95% CI | *w_i_*+ | *N* | *P* |
| --- | --- | --- | --- | --- | --- | --- |
| (Intercept) | 1.106 | 0.047 | [1.01E+00, 1.20E+00] |  |  | <2.00E-16 *** |
| Area closed patches (mean) | -0.058 | 0.066 | [-2.13E-01, 2.09E-02] | 0.61 | 3 | 0.378 |
| **Riparian habitat** | **0.164** | **0.048** | **[6.88E-02, 2.59E-01]** | **1** | **6** | **7.3E-4 ***** |
| **Slope >30 (area)** | **0.069** | **0.050** | **[3.16E-03, 1.68E-01]** | **0.81** | **4** | **0.174** |
| **Water bodies** | **0.261** | **0.049** | **[1.64E-01, 3.58E-01]** | **1** | **6** | **1.0E-7 ***** |
| Open patches richness | 0.013 | 0.035 | [-5.68E-02, 1.50E-01] | 0.29 | 2 | 0.706 |

**Table S3 –** Summary results of average models relating total bat activity to landscape composition and structure variables. Models were built separately for each phenological period (Pregnancy, Lactation, Post-Lactation) and for data aggregated across periods (Annual). In each case, separate models were also built for landscape composition, landscape structure, and the combination of landscape composition and structure variables. For each variable we provide the estimate of the regression coefficient (B), its standard error (SE) and 95% confidence interval (95%CI), the sum of Akaike weights (*w_i_*+), the number of models in which the variable was included (*N*), and the significance level (*P*). *** *P* < 0.001; ** *P* < 0.01; * *P* < 0.05. Variables with *w_i_*+ >0.70 are highlighted in bold

**Landscape composition models**

**Pregnancy**

| Variables | B | SE | 95% CI | *w_i_*+ | *N* | *P* |
| --- | --- | --- | --- | --- | --- | --- |
| (Intercept) | 3.458 | 0.172 | [3.11E+00, 3.80E+00] |  |  | < 2.0E-16 *** |
| Mediterranean forest | 0.042 | 0.223 | [-6.03E-01, 8.46E-01] | 0.35 | 16 | 0.852 |
| Orchards | 0.215 | 0.385 | [-4.32E-01, 1.36E+00] | 0.46 | 17 | 0.579 |
| **Riparian habitat** | **0.389** | **0.183** | **[8.72E-02, 7.36E-01]** | **0.94** | **30** | **0.037 *** |
| Shrublands | 0.146 | 0.336 | [-5.41E-01, 1.20E+00] | 0.44 | 20 | 0.665 |
| **Water bodies** | **0.817** | **0.230** | **[3.57E-01, 1.28E+00]** | **1** | **39** | **5.0E-4 ***** |
| Arable land | 0.133 | 0.254 | [-2.80E-01, 9.50E-01] | 0.4 | 16 | 0.605 |
| **Conifers** | **-0.188** | **0.236** | **[-7.41E-01, 2.18E-01]** | **0.72** | **21** | **0.431** |

**Lactation**

| Variables | B | SE | 95% CI | *w_i_*+ | *N* | *P* |
| --- | --- | --- | --- | --- | --- | --- |
| (Intercept) | 3.631 | 0.208 | [3.21E+00, 4.05E+00] |  |  | < 2.0E-16 *** |
| Mediterranean forest | 0.057 | 1.247 | [-3.62E+00, 3.88E+00] | 0.43 | 19 | 0.964 |
| Orchards | -0.171 | 1.585 | [-4.60E+00, 3.97E+00] | 0.53 | 18 | 0.915 |
| **Riparian habitat** | **0.581** | **0.232** | **[1.86E-01, 1.02E+00]** | **0.96** | **30** | **0.014 *** |
| Shrublands | 0.320 | 1.717 | [-3.80E+00, 4.85E+00] | 0.61 | 20 | 0.854 |
| **Water bodies** | **0.998** | **0.278** | **[5.02E-01, 1.52E+00]** | **0.99** | **34** | **4.1E-4 ***** |
| **Arable land** | **-0.252** | **0.987** | **[-2.75E+00, 1.99E+00]** | **0.66** | **20** | **0.801** |
| Conifers | 0.000 | 1.135 | [-4.16E+00, 4.16E+00] | 0.29 | 15 | 1.000 |

**Post-lactation**

| Variables | B | SE | 95% CI | *w_i_*+ | *N* | *P* |
| --- | --- | --- | --- | --- | --- | --- |
| (Intercept) | 3.157 | 0.220 | [2.71E+00, 3.60E+00] |  |  | <2.0E-16 *** |
| Mediterranean forest | 0.028 | 0.132 | [-3.83E-01, 5.95E-01] | 0.26 | 19 | 0.839 |
| Orchards | 0.004 | 0.134 | [-5.17E-01, 5.50E-01] | 0.26 | 19 | 0.976 |
| Riparian habitat | 0.094 | 0.185 | [-2.19E-01, 7.03E-01] | 0.39 | 22 | 0.616 |
| Shrublands | -0.022 | 0.124 | [-5.54E-01, 3.82E-01] | 0.26 | 19 | 0.862 |
| **Water bodies** | **1.023** | **0.170** | **[6.79E-01, 1.37E+00]** | **1** | **48** | **<2.0E-16 ***** |
| **Arable land** | **-0.389** | **0.359** | **[-1.15E+00, -3.47E-02]** | **0.66** | **26** | **0.285** |
| Conifers | 0.024 | 0.116 | [-3.36E-01, 5.20E-01] | 0.26 | 19 | 0.840 |

**Annual**

| Variables | B | SE | 95% CI | *w_i_*+ | *N* | *P* |
| --- | --- | --- | --- | --- | --- | --- |
| (Intercept) | 3.429 | 0.101 | [3.23E+00, 3.63E+00] |  |  | < 2E-16 *** |
| Mediterranean forest | 0.144 | 0.247 | [-2.92E-01, 8.19E-01] | 0.55 | 15 | 0.560 |
| Orchards | 0.087 | 0.291 | [-6.24E-01, 1.06E+00] | 0.4 | 13 | 0.765 |
| **Riparian habitat** | **0.349** | **0.111** | **[1.30E-01, 5.69E-01]** | **1** | **29** | **1.78E-3 **** |
| Shrublands | 0.177 | 0.333 | [-4.40E-01, 1.12E+00] | 0.52 | 14 | 0.595 |
| **Water bodies** | **0.920** | **0.113** | **[6.96E-01, 1.14E+00]** | **1** | **29** | **< 2E-16 ***** |
| Arable land | -0.077 | 0.191 | [-6.15E-01, 3.32E-01] | 0.54 | 16 | 0.689 |
| Conifers | -0.013 | 0.205 | [-6.30E-01, 5.72E-01] | 0.45 | 14 | 0.950 |

**Landscape structure models**

**Pregnancy**

| Variables | B | SE | 95% CI | *w_i_*+ | *N* | *P* |
| --- | --- | --- | --- | --- | --- | --- |
| (Intercept) | 3.355 | 0.170 | [3.01E+00, 3.70E+00] |  |  | <2E-16 *** |
| Altitude (stdev) | -0.038 | 0.129 | [-5.52E-01, 2.97E-01] | 0.3 | 1857 | 0.774 |
| **Area closed patches (mean)** | **-0.351** | **0.232** | **[-7.77E-01, -1.04E-01]** | **0.8** | **2813** | **0.134** |
| Edge density open patches | 0.031 | 0.143 | [-3.94E-01, 6.11E-01] | 0.29 | 1854 | 0.832 |
| Northness (median) | -0.055 | 0.152 | [-6.30E-01, 2.83E-01] | 0.32 | 1879 | 0.720 |
| Slope >30 (area) | -0.040 | 0.117 | [-4.97E-01, 2.34E-01] | 0.3 | 1805 | 0.738 |
| Open patches richness | 0.005 | 0.103 | [-3.77E-01, 4.11E-01] | 0.27 | 1809 | 0.965 |
| Number closed patches | 0.011 | 0.085 | [-2.79E-01, 3.61E-01] | 0.27 | 1792 | 0.898 |
| Edge density closed patches | -0.034 | 0.093 | [-3.89E-01, 1.72E-01] | 0.31 | 1787 | 0.721 |
| Eastness (median) | -0.004 | 0.131 | [-5.25E-01, 4.92E-01] | 0.27 | 1756 | 0.974 |
| Closed patches richness | 0.007 | 0.113 | [-4.09E-01, 4.63E-01] | 0.27 | 1773 | 0.95 |
| Slope (range) | 0.018 | 0.114 | [-3.58E-01, 4.90E-01] | 0.27 | 1791 | 0.878 |
| Area open patches (mean) | 0.199 | 0.363 | [-3.61E-01, 1.34E+00] | 0.41 | 2124 | 0.587 |
| Number open patches | 0.052 | 0.188 | [-4.45E-01, 7.70E-01] | 0.32 | 1890 | 0.785 |

**Lactation**

| Variables | B | SE | 95% CI | *w_i_*+ | *N* | *P* |
| --- | --- | --- | --- | --- | --- | --- |
| (Intercept) | 3.026 | 0.184 | [2.66E+00, 3.39E+00] |  |  | <2.0E-16 *** |
| Altitude (stdev) | 0.038 | 0.130 | [-2.98E-01, 5.60E-01] | 0.29 | 811 | 0.774 |
| Area closed patches (mean) | 0.125 | 0.205 | [-2.05E-01, 7.13E-01] | 0.49 | 919 | 0.547 |
| Edge density open patches | 0.033 | 0.154 | [-4.20E-01, 6.39E-01] | 0.3 | 922 | 0.833 |
| Northness (median) | -0.055 | 0.114 | [-4.55E-01, 1.24E-01] | 0.33 | 848 | 0.634 |
| **Slope >30 (area)** | **1.076** | **0.223** | **[6.32E-01, 1.52E+00]** | **1** | **1942** | **2.0E-06 ***** |
| Open patches richness | 0.017 | 0.118 | [-3.76E-01, 5.02E-01] | 0.27 | 815 | 0.886 |
| **Number closed patches** | **0.500** | **0.490** | **[-8.48E-02, 1.58E+00]** | **0.67** | **1164** | **0.312** |
| Edge density closed patches | -0.219 | 0.444 | [-1.68E+00, 5.42E-01] | 0.38 | 890 | 0.625 |
| Eastness (median) | -0.068 | 0.126 | [-4.81E-01, 1.11E-01] | 0.37 | 854 | 0.595 |
| Closed patches richness | -0.085 | 0.216 | [-8.59E-01, 3.69E-01] | 0.35 | 811 | 0.697 |
| Slope (range) | -0.066 | 0.132 | [-5.03E-01, 1.57E-01] | 0.38 | 886 | 0.621 |
| Area open patches (mean) | 0.199 | 0.652 | [-1.42E+00, 2.53E+00] | 0.36 | 959 | 0.763 |
| **Number open patches** | **0.926** | **0.405** | **[2.50E-01, 1.69E+00]** | **0.96** | **1648** | **0.024 *** |

**Post-lactation**

| Variables | B | SE | 95% CI | *w_i_*+ | *N* | *P* |
| --- | --- | --- | --- | --- | --- | --- |
| (Intercept) | 3.664 | 0.218 | [3.22E+00, 4.10E+00] |  |  | < 2.0E-16 *** |
| Altitude (stdev) | -0.088 | 0.242 | [-9.88E-01, 4.58E-01] | 0.33 | 1125 | 0.722 |
| Area closed patches (mean) | -0.136 | 0.228 | [-8.30E-01, 1.78E-01] | 0.42 | 1273 | 0.556 |
| Edge density open patches | -0.075 | 0.216 | [-8.93E-01, 4.30E-01] | 0.32 | 1121 | 0.734 |
| Northness (median) | 0.014 | 0.168 | [-5.97E-01, 6.99E-01] | 0.27 | 1057 | 0.937 |
| Slope >30 (area) | 0.226 | 0.301 | [-1.85E-01, 1.01E+00] | 0.54 | 1402 | 0.460 |
| **Open patches richness** | **0.956** | **0.198** | **[5.56E-01, 1.36E+00]** | **1** | **2656** | **2.8E-06 ***** |
| Number closed patches | 0.014 | 0.167 | [-5.89E-01, 6.94E-01] | 0.27 | 1071 | 0.934 |
| Edge density closed patches | 0.140 | 0.254 | [-2.70E-01, 9.31E-01] | 0.42 | 1334 | 0.587 |
| Eastness (median) | -0.023 | 0.135 | [-5.94E-01, 4.19E-01] | 0.26 | 1049 | 0.867 |
| Closed patches richness | -0.040 | 0.172 | [-7.28E-01, 4.60E-01] | 0.3 | 1112 | 0.822 |
| Slope (range) | -0.259 | 0.330 | [-1.10E+00, 1.68E-01] | 0.56 | 1345 | 0.439 |
| Area open patches (mean) | 0.025 | 0.101 | [-2.62E-01, 4.35E-01] | 0.29 | 1064 | 0.808 |
| Number open patches | -0.030 | 0.143 | [-6.25E-01, 4.05E-01] | 0.28 | 1031 | 0.836 |

**Annual**

| Variables | B | SE | 95% CI | *w_i_*+ | *N* | *P* |
| --- | --- | --- | --- | --- | --- | --- |
| (Intercept) | 3.508 | 0.111 | [3.29E+00, 3.73E+00] |  |  | < 2.0E-16 *** |
| Altitude (stdev) | -0.082 | 0.139 | [-5.09E-01, 7.83E-02] | 0.38 | 773 | 0.558 |
| **Area closed patches (mean)** | -0.020 | 0.075 | [-3.17E-01, 1.85E-01] | 0.29 | 746 | 0.797 |
| Edge density open patches | 0.109 | 0.145 | [-7.38E-02, 4.84E-01] | 0.53 | 883 | 0.453 |
| Northness (median) | -0.007 | 0.058 | [-2.47E-01, 1.94E-01] | 0.26 | 712 | 0.908 |
| **Slope >30 (area)** | **0.397** | **0.143** | **[1.32E-01, 6.72E-01]** | **0.99** | **1631** | **5.9E-3 **** |
| **Open patches richness** | **0.487** | **0.127** | **[2.37E-01, 7.37E-01]** | **1** | **1727** | **1.4E-4 ***** |
| Number closed patches | 0.024 | 0.088 | [-2.08E-01, 3.68E-01] | 0.3 | 751 | 0.787 |
| Edge density closed patches | -0.019 | 0.075 | [-3.20E-01, 1.88E-01] | 0.29 | 721 | 0.802 |
| Eastness (median) | 0.003 | 0.060 | [-2.18E-01, 2.42E-01] | 0.26 | 710 | 0.959 |
| Closed patches richness | -0.022 | 0.089 | [-3.68E-01, 2.22E-01] | 0.3 | 743 | 0.805 |
| Slope (range) | 0.034 | 0.085 | [-1.40E-01, 3.46E-01] | 0.33 | 744 | 0.694 |
| Area open patches (mean) | 0.159 | 0.219 | [-8.10E-02, 7.40E-01] | 0.48 | 831 | 0.470 |
| Number open patches | 0.129 | 0.188 | [-1.17E-01, 6.40E-01] | 0.49 | 847 | 0.494 |

**Global models**

**Pregnancy**

| Variables | B | SE | 95% CI | *w_i_*+ | *N* | *P* |
| --- | --- | --- | --- | --- | --- | --- |
| (Intercept) | 3.197 | 0.159 | [2.88E+00, 3.52E+00] |  |  | < 2.0E-16 *** |
| **Riparian habitat** | **0.289** | **0.173** | **[2.79E-02, 6.22E-01]** | **0.89** | **4** | **0.099 .** |
| **Water bodies** | **0.699** | **0.216** | **[2.67E-01, 1.13E+00]** | **1** | **6** | **1.5E-3 **** |
| Conifers | -0.169 | 0.164 | [-5.26E-01, -4.25E-03] | 0.64 | 3 | 0.310 |
| Area closed patches (mean) | -0.127 | 0.174 | [-5.87E-01, 9.19E-02] | 0.51 | 3 | 0.468 |

**Lactation**

| Variables | B | SE | 95% CI | *w_i_*+ | *N* | *P* |
| --- | --- | --- | --- | --- | --- | --- |
| (Intercept) | 3.030 | 0.167 | [2.70E+00, 3.36E+00] |  |  | < 2.0E-16 *** |
| Riparian habitat | 0.068 | 0.169 | [-2.79E-01, 6.84E-01] | 0.33 | 6 | 0.693 |
| **Slope >30 (area)** | **0.870** | **0.227** | **[4.17E-01, 1.32E+00]** | **1** | **14** | **1.7E-4 ***** |
| Water bodies | 0.178 | 0.223 | [-1.35E-01, 7.33E-01] | 0.59 | 8 | 0.430299 |
| **Number open patches** | **0.721** | **0.216** | **[2.90E-01, 1.15E+00]** | **1** | **14** | **1.0E-3 **** |
| **Arable land** | **-0.274** | **0.184** | **[-6.21E-01, -5.78E-02]** | **0.81** | **8** | **0.141** |
| Number closed patches | 0.106 | 0.226 | [-2.99E-01, 8.75E-01] | 0.37 | 7 | 0.642 |

**Post-lactation**

| Variables | B | SE | 95% CI | *w_i_*+ | *N* | *P* |
| --- | --- | --- | --- | --- | --- | --- |
| (Intercept) | 3.161 | 0.210 | [2.74E+00, 3.58E+00] |  |  | < 2E-16 *** |
| **Water bodies** | **0.736** | **0.265** | **[2.07E-01, 1.26E+00]** | **1** | **4** | **6.39E-3 **** |
| Open patches richness | 0.307 | 0.268 | [2.51E-02, 8.63E-01] | 0.69 | 2 | 0.257 |
| **Arable land** | **-0.583** | **0.377** | **[-1.28E+00, -1.70E-01]** | **0.81** | **2** | **0.127** |

**Annual**

| Variables | B | SE | 95% CI | *w_i_*+ | *N* | *P* |
| --- | --- | --- | --- | --- | --- | --- |
| (Intercept) | 3.313 | 0.101 | [3.11E+00, 3.51E+00] |  |  | < 2.0E-16 *** |
| **Riparian habitat** | **0.381** | **0.105** | **[1.74E-01, 5.89E-01]** | **1** | **3** | **3.2E-4 ***** |
| Slope >30 (area) | 0.105 | 0.116 | [-4.57E-02, 3.74E-01] | 0.64 | 2 | 0.367 |
| **Water bodies** | **0.920** | **0.128** | **[6.69E-01, 1.17E+00]** | **1** | **3** | **< 2.0E-16 ***** |
| Open patches richness | 0.033 | 0.081 | [-9.53E-02, 3.59E-01] | 0.25 | 1 | 0.685 |

**Table S4 –** Summary results of average models relating total bat activity, excluding *Pipistrellus pipistrellus* and Non Identified bat passes, to landscape composition and structure variables. Models were built separately for each phenological period (Pregnancy, Lactation, Post-Lactation) and for data aggregated across periods (Annual). In each case, separate models were also built for landscape composition, landscape structure, and the combination of landscape composition and structure variables. For each variable we provide the estimate of the regression coefficient (B), its standard error (SE) and 95% confidence interval (95%CI), the sum of Akaike weights (*w_i_*+), the number of models in which the variable was included (*N*), and the significance level (*P*). *** *P* < 0.001; ** *P* < 0.01; * *P* < 0.05. Variables with *w_i_*+ >0.70 are highlighted in bold

**Landscape composition**

**Pregnancy**

| Variables | B | SE | 95% CI | *w_i_*+ | *N* | *P* |
| --- | --- | --- | --- | --- | --- | --- |
| (Intercept) | 2.340 | 0.206 | [1.93E+00, 2.75E+00] | 0.29 | 41 | <2.0E-16 *** |
| Arable land | -0.048 | 0.161 | [-6.94E-01, 3.64E-01] | 0.32 | 41 | 0.769 |
| Conifers | -0.045 | 0.119 | [-4.89E-01, 2.11E-01] | 0.27 | 40 | 0.709 |
| Riparian habitat | -0.016 | 0.105 | [-4.49E-01, 3.30E-01] | 0.5 | 47 | 0.880 |
| Water bodies | 0.177 | 0.243 | [-1.18E-01, 8.25E-01] | 0.31 | 42 | 0.471 |
| Shrublands | -0.022 | 0.149 | [-5.90E-01, 4.49E-01] | 0.32 | 43 | 0.885 |
| Mediterranean forest | 0.054 | 0.143 | [-2.55E-01, 5.90E-01] | 0.47 | 46 | 0.710 |
| Orchards | 0.164 | 0.262 | [-2.18E-01, 9.22E-01] | 0.29 | 41 | 0.536 |

**Lactation**

| Variables | B | SE | 95% CI | *w_i_*+ | *N* | *P* |
| --- | --- | --- | --- | --- | --- | --- |
| (Intercept) | 2.426 | 0.179 | [2.07E+00, 2.78E+00] |  |  | < 2.0E-16 *** |
| **Arable land** | **-0.252** | **0.442** | **[-1.33E+00, 6.02E-01]** | **0.70** | **31** | **0.569** |
| Conifers | 0.064 | 0.491 | [-1.35E+00, 1.67E+00] | 0.40 | 25 | 0.896 |
| Riparian habitat | 0.210 | 0.228 | [-3.98E-02, 7.40E-01] | 0.60 | 29 | 0.363 |
| **Water bodies** | **0.786** | **0.197** | **[4.31E-01, 1.16E+00]** | **0.99** | **52** | **8.6e-05 ***** |
| Shrublands | 0.026 | 0.713 | [-2.21E+00, 2.35E+00] | 0.38 | 27 | 0.971 |
| Mediterranean forest | -0.072 | 0.521 | [-2.09E+00, 1.60E+00] | 0.29 | 23 | 0.891 |
| Orchards | -0.075 | 0.653 | [-2.53E+00, 2.04E+00] | 0.30 | 23 | 0.909 |

**Post-lactation**

| Variables | B | SE | 95% CI | *w_i_*+ | *N* | *P* |
| --- | --- | --- | --- | --- | --- | --- |
| (Intercept) | 2.303 | 0.225 | [1.85E+00, 2.76E+00] |  |  | <2.0E-16 *** |
| **Arable land** | **-0.579** | **0.431** | **[-1.41E+00, -1.19E-01]** | **0.76** | **26** | **0.185** |
| Conifers | -0.119 | 0.228 | [-8.52E-01, 2.53E-01] | 0.40 | 18 | 0.605 |
| Riparian habitat | 0.003 | 0.112 | [-4.48E-01, 4.74E-01] | 0.24 | 17 | 0.979 |
| **Water bodies** | **0.850** | **0.166** | **[5.15E-01, 1.19E+00]** | **1** | **46** | **7.0E-07 ***** |
| Shrublands | 0.070 | 0.255 | [-6.05E-01, 1.07E+00] | 0.30 | 19 | 0.786 |
| Mediterranean forest | 0.050 | 0.204 | [-5.24E-01, 8.77E-01] | 0.28 | 20 | 0.808 |
| Orchards | 0.061 | 0.252 | [-6.47E-01, 1.06E+00] | 0.29 | 20 | 0.81 |

**Annual**

| Variables | B | SE | 95% CI | *w_i_*+ | *N* | *P* |
| --- | --- | --- | --- | --- | --- | --- |
| (Intercept) | 2.377 | 0.102 | [2.17E+00, 2.58E+00] |  |  | <2.0E-16 *** |
| **Arable land** | **-0.183** | **0.156** | **[-5.04E-01, 1.83E-04]** | **0.73** | **29** | **0.243** |
| Conifers | -0.032 | 0.111 | [-4.18E-01, 2.46E-01] | 0.37 | 21 | 0.774 |
| Riparian habitat | 0.031 | 0.077 | [-1.21E-01, 3.17E-01] | 0.32 | 21 | 0.689 |
| **Water bodies** | **0.733** | **0.108** | **[5.20E-01, 9.46E-01]** | **1** | **50** | **<2.0E-16 ***** |
| Shrublands | 0.085 | 0.175 | [-2.32E-01, 6.40E-01] | 0.41 | 25 | 0.629 |
| Mediterranean forest | 0.025 | 0.112 | [-2.98E-01, 4.63E-01] | 0.3 | 21 | 0.826 |
| Orchards | 0.047 | 0.149 | [-3.11E-01, 5.90E-01] | 0.34 | 24 | 0.751 |

**Landscape structure models**

**Pregnancy**

| Variables | B | SE | 95% CI | *w_i_*+ | *N* | *P* |
| --- | --- | --- | --- | --- | --- | --- |
| (Intercept) | 2.211 | 0.192 | [1.83E+00, 2.60E+00] |  |  | <2.0E-16 *** |
| Altitude (stdev) | -0.105 | 0.239 | [-9.34E-01, 3.52E-01] | 0.36 | 2264 | 0.663 |
| Area closed patches (mean) | -0.084 | 0.163 | [-6.20E-01, 1.81E-01] | 0.38 | 2419 | 0.611 |
| Edge density open patches | 0.187 | 0.288 | [-2.13E-01, 1.00E+00] | 0.47 | 2556 | 0.520 |
| Northness (median) | 0.060 | 0.183 | [-3.81E-01, 7.76E-01] | 0.3 | 2234 | 0.749 |
| Slope >30 (area) | 0.193 | 0.258 | [-1.30E-01, 8.66E-01] | 0.52 | 2812 | 0.459 |
| Open patches richness | -0.013 | 0.115 | [-4.83E-01, 3.85E-01] | 0.27 | 2157 | 0.910 |
| **Number closed patches** | **0.202** | **0.193** | **[-8.41E-02, 6.28E-01]** | **0.74** | **3328** | **0.305** |
| Edge density closed patches | -0.028 | 0.110 | [-4.60E-01, 2.76E-01] | 0.31 | 2180 | 0.802 |
| Eastness (median) | -0.056 | 0.183 | [-7.66E-01, 4.00E-01] | 0.31 | 2248 | 0.761 |
| Closed patches richness | 0.003 | 0.214 | [-7.14E-01, 7.31E-01] | 0.35 | 2385 | 0.990 |
| Slope (range) | 0.075 | 0.178 | [-2.82E-01, 7.03E-01] | 0.36 | 2323 | 0.678 |
| Area open patches (mean) | 0.264 | 0.394 | [-2.54E-01, 1.37E+00] | 0.47 | 2483 | 0.508 |
| Number open patches | -0.112 | 0.243 | [-9.29E-01, 3.41E-01] | 0.38 | 2337 | 0.648 |

**Lactation**

| Variables | B | SE | 95% CI | *w_i_*+ | *N* | *P* |
| --- | --- | --- | --- | --- | --- | --- |
| (Intercept) | 2.144 | 0.177 | [1.79E+00, 2.50E+00] |  |  | < 2.0E-16 *** |
| Altitude (stdev) | 0.049 | 0.143 | [-2.86E-01, 5.87E-01] | 0.32 | 920 | 0.737 |
| **Area closed patches (mean)** | **0.286** | **0.243** | **[-5.85E-02, 7.96E-01]** | **0.78** | **1063** | **0.245** |
| Edge density open patches | 0.294 | 0.292 | [-5.13E-02, 9.39E-01] | 0.66 | 1054 | 0.317 |
| Northness (median) | -0.029 | 0.084 | [-3.54E-01, 1.61E-01] | 0.3 | 790 | 0.731 |
| **Slope >30 (area)** | **0.588** | **0.218** | **[2.02E-01, 1.00E+00]** | **0.98** | **1654** | **7.9E-3 **** |
| Open patches richness | -0.013 | 0.113 | [-4.67E-01, 3.75E-01] | 0.28 | 815 | 0.911 |
| **Number closed patches** | **1.137** | **0.404** | **[3.60E-01, 1.93E+00]** | **0.99** | **1803** | **5.6E-3 **** |
| Edge density closed patches | -0.235 | 0.412 | [-1.51E+00, 3.61E-01] | 0.41 | 860 | 0.572 |
| Eastness (median) | -0.005 | 0.074 | [-3.02E-01, 2.66E-01] | 0.27 | 770 | 0.949 |
| Closed patches richness | -0.211 | 0.275 | [-9.21E-01, 1.31E-01] | 0.53 | 955 | 0.448 |
| Slope (range) | -0.089 | 0.143 | [-5.03E-01, 1.20E-01] | 0.46 | 864 | 0.538 |
| Area open patches (mean) | -0.228 | 0.570 | [-2.08E+00, 9.98E-01] | 0.42 | 1023 | 0.692 |
| Number open patches | 0.338 | 0.372 | [-7.02E-02, 1.20E+00] | 0.6 | 1014 | 0.367 |

**Post-lactation**

| Variables | B | SE | 95% CI | *w_i_*+ | *N* | *P* |
| --- | --- | --- | --- | --- | --- | --- |
| (Intercept) | 2.792 | 0.216 | [2.36E+00, 3.23E+00] |  |  | < 2.0E-16 *** |
| Altitude (stdev) | -0.029 | 0.185 | [-7.63E-01, 5.65E-01] | 0.29 | 1092 | 0.877 |
| **Area closed patches (mean)** | **-0.373** | **0.298** | **[-9.67E-01, -5.25E-02]** | **0.73** | **1580** | **0.217** |
| Edge density open patches | -0.029 | 0.199 | [-8.04E-01, 6.11E-01] | 0.3 | 1104 | 0.886 |
| Northness (median) | 0.040 | 0.170 | [-4.56E-01, 7.49E-01] | 0.28 | 1042 | 0.816 |
| Slope >30 (area) | 0.099 | 0.217 | [-3.15E-01, 8.36E-01] | 0.38 | 1214 | 0.654 |
| **Open patches richness** | **0.781** | **0.193** | **[3.91E-01, 1.17E+00]** | **1** | **2641** | **8.8E-05 ***** |
| Number closed patches | -0.080 | 0.212 | [-8.35E-01, 3.94E-01] | 0.36 | 1136 | 0.712 |
| Edge density closed patches | 0.064 | 0.196 | [-4.15E-01, 8.10E-01] | 0.32 | 1100 | 0.75 |
| Eastness (median) | -0.002 | 0.124 | [-5.00E-01, 4.84E-01] | 0.26 | 1037 | 0.987 |
| Closed patches richness | -0.114 | 0.216 | [-7.87E-01, 2.65E-01] | 0.44 | 1295 | 0.603 |
| Slope (range) | -0.264 | 0.321 | [-1.07E+00, 1.46E-01] | 0.57 | 1373 | 0.418 |
| Area open patches (mean) | 0.030 | 0.104 | [-2.43E-01, 4.41E-01] | 0.3 | 1120 | 0.777 |
| Number open patches | -0.026 | 0.152 | [-6.39E-01, 4.58E-01] | 0.29 | 1051 | 0.867 |

**Annual**

| Variables | B | SE | 95% CI | *w_i_*+ | *N* | *P* |
| --- | --- | --- | --- | --- | --- | --- |
| (Intercept) | 2.484 | 0.109 | [2.27E+00, 2.70E+00] |  |  | < 2.0E-16 *** |
| Altitude (stdev) | -0.033 | 0.097 | [-4.07E-01, 1.91E-01] | 0.3 | 634 | 0.736 |
| Area closed patches (mean) | -0.001 | 0.068 | [-2.62E-01, 2.53E-01] | 0.27 | 604 | 0.987 |
| Edge density open patches | 0.168 | 0.181 | [-4.59E-02, 5.82E-01] | 0.63 | 847 | 0.355 |
| Northness (median) | 0.010 | 0.060 | [-1.81E-01, 2.57E-01] | 0.27 | 590 | 0.867 |
| **Slope >30 (area)** | **0.367** | **0.135** | **[1.22E-01, 6.24E-01]** | **0.98** | **1372** | **6.7E-3 **** |
| **Open patches richness** | **0.413** | **0.126** | **[1.66E-01, 6.60E-01]** | **1** | **1474** | **1.1E-3 **** |
| **Number closed patches** | **0.227** | **0.192** | **[-8.76E-03, 6.20E-01]** | **0.74** | **839** | **0.237** |
| Edge density closed patches | -0.010 | 0.070 | [-2.93E-01, 2.23E-01] | 0.27 | 608 | 0.892 |
| Eastness (median) | -0.031 | 0.079 | [-3.26E-01, 1.24E-01] | 0.31 | 618 | 0.694 |
| **Closed patches richness** | **-0.239** | **0.202** | **[-6.53E-01, 1.32E-02]** | **0.75** | **937** | **0.237** |
| Slope (range) | 0.017 | 0.070 | [-1.79E-01, 2.98E-01] | 0.29 | 601 | 0.808 |
| Area open patches (mean) | 0.147 | 0.174 | [-3.31E-02, 5.66E-01] | 0.55 | 764 | 0.400 |
| Number open patches | -0.010 | 0.118 | [-4.36E-01, 3.75E-01] | 0.32 | 652 | 0.934 |

**Figure S2 –** Standard error of species richness and activity projections within the study area for each phenological stage (Pregnancy, Lactation and Post-lactation) and for the data set pooling the yearly data (Annual). Species richness values refer to 15 minutes sampling while total activity refers to bat passes.min^-1^
